# Genetic evidence of tri-genealogy hypothesis on the origin of ethnic minorities in Yunnan

**DOI:** 10.1186/s12915-022-01367-3

**Published:** 2022-07-21

**Authors:** Zhaoqing Yang, Hao Chen, Yan Lu, Yang Gao, Hao Sun, Jiucun Wang, Li Jin, Jiayou Chu, Shuhua Xu

**Affiliations:** 1 Department of Medical Genetics, Institute of Medical Biology, Chinese Academy of Medical Sciences, Kunming, 650118 China; 2 Key Laboratory of Computational Biology, Shanghai Institute of Nutrition and Health, University of Chinese Academy of Sciences, Chinese Academy of Sciences, Shanghai, 200031 China; 3 State Key Laboratory of Genetic Engineering, Collaborative Innovation Center for Genetics and Development, Center for Evolutionary Biology, School of Life Sciences, Fudan University, Shanghai, 200438 China; 4 Human Phenome Institute, Zhangjiang Fudan International Innovation Center, and Ministry of Education Key Laboratory of Contemporary Anthropology, Fudan University, Shanghai, 201203 China; 5 Department of Liver Surgery and Transplantation Liver Cancer Institute, Zhongshan Hospital, Fudan University, Shanghai, 200032 China; 6 Center for Excellence in Animal Evolution and Genetics, Chinese Academy of Sciences, Kunming, 650223 China

**Keywords:** Yunnan, Ethnic minorities, Population history, Tri-genealogy hypothesis, Local adaptation

## Abstract

**Background:**

Yunnan is located in Southwest China and consists of great cultural, linguistic, and genetic diversity. However, the genomic diversity of ethnic minorities in Yunnan is largely under-investigated. To gain insights into population history and local adaptation of Yunnan minorities, we analyzed 242 whole-exome sequencing data with high coverage (~ 100–150 ×) of Yunnan minorities representing Achang, Jingpo, Dai, and Deang, who were linguistically assumed to be derived from three ancient lineages (the tri-genealogy hypothesis), i.e., Di-Qiang, Bai-Yue, and Bai-Pu.

**Results:**

Yunnan minorities show considerable genetic differences. Di-Qiang populations likely migrated from the Tibetan area about 6700 years ago. Genetic divergence between Bai-Yue and Di-Qiang was estimated to be 7000 years, and that between Bai-Yue and Bai-Pu was estimated to be 5500 years. Bai-Pu is relatively isolated, but gene flow from surrounding Di-Qiang and Bai-Yue populations was also found. Furthermore, we identified genetic variants that are differentiated within Yunnan minorities possibly due to the living circumstances and habits. Notably, we found that adaptive variants related to malaria and glucose metabolism suggest the adaptation to thalassemia and G6PD deficiency resulting from malaria resistance in the Dai population.

**Conclusions:**

We provided genetic evidence of the tri-genealogy hypothesis as well as new insights into the genetic history and local adaptation of the Yunnan minorities.

**Supplementary Information:**

The online version contains supplementary material available at 10.1186/s12915-022-01367-3.

## Background

**Fig. 1:**
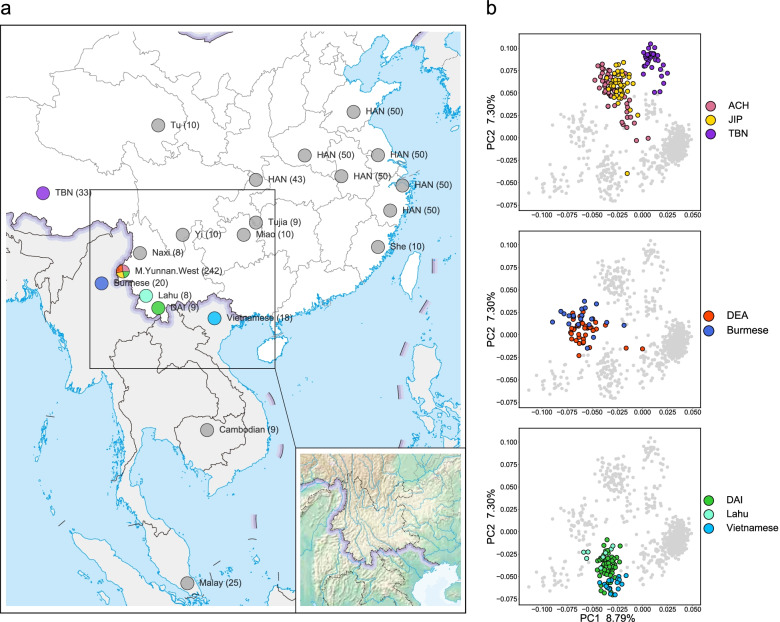
Sample information and PCA of Global Panel C. **a** Geographic location of samples in Global Panel C. The circle color of each population on map corresponds to the dot color of PC plots in **b**. **b** M.Yunnan.West and corresponding populations with close affinities in the PCA of Global Panel C, using a total number of 17,101 SNPs in 709 individuals

Located in Southwest China, Yunnan is a territory with a diversified ecological environment, a complex terrain of which over 90% is covered by mountains and hills, and it borders multiple countries in mainland Southeast Asia (Fig. 1a), which have given birth to rich human genetic and cultural diversity [1, 2]. With various ethnic minorities living in the same region, in general, population admixture can lead to frequent genetic exchanges among different ethnic groups [3]. Nonetheless, due to the complexity of mountainous landforms, Yunnan serves as a natural barrier for different ethnic groups to be isolated. As a result, these minorities are considered to have retained their cultural traditions and genetic patterns along with their histories. However, the genetic origin and history of most minorities living in Yunnan are still opaque. A comparison of the genetic backgrounds and evolutionary histories of Yunnan minorities is required to uncover the human genetic diversity [4].

Archeological records have shown that humans settled in Yunnan in the late Paleolithic period about 10,000 years ago (ya), and the Neolithic culture in Yunnan developed prosperously with rice culture around 5000 ya [5, 6]. In addition, a splendid Bronze Age civilization of Yunnan around 4000–3500 ya was derived from the Haimenkou Relic Site. The unearthed bronzes are distinguished from those from the Central Plains based on their advanced craftsmanship and strong ethnic characteristics [5]. These findings provide robust evidence that indigenous people living in Yunnan were distinct from inland people in the past. However, ethnological and linguistic studies have proposed the tri-genealogy hypothesis, which states that most ethnic minorities living in Yunnan have three different origins and histories. Generally speaking, the present-day diverse minorities in Yunnan are mainly derived from three ancient lineages, i.e., Bai-Yue, Bai-Pu, and Di-Qiang [7]. The Bai-Yue and Bai-Pu were southern indigenous groups speaking Tai-Kadai (also known as Kra-Dai) and Austroasiatic languages, respectively [7–9]. The Di-Qiang are northern immigrants speaking Tibeto-Burman languages who migrated from the Upper-Middle Yellow River Basin through the Tibetan-Yi corridor to Northwestern Yunnan because of the expansion of the Qin dynasty around 2700 ya [1, 8, 10–15]. Furthermore, previous Y-chromosomal analyses have indicated that the present Yunnan minorities who speak Tibeto-Burman languages are the result of admixture between Neolithic immigrants and local populations based on the relatively high frequency of the NRY haplogroups D-M174 and O-M95 [13, 16, 17]. The geographic distribution of minorities in Yunnan belonging to these three lineages also shows a certain pattern: (i) Di-Qiang mainly live in Northwestern Yunnan, (ii) Bai-Yue in Southwestern Yunnan and near the border between Eastern Yunnan and the Guangxi Zhuang Autonomous Region, and (iii) Bai-Pu in Western Yunnan, which borders with Myanmar (Additional file 1: Fig. S1). Coincidentally, minorities living in Western Yunnan are included in these three ancient lineages (Additional file 1: Fig. S1) with different historical cultures (Additional file 2), making it an appropriate region to investigate different Yunnan ethnic minorities.

Exome sequencing technology is a popular approach to generating high-coverage data to discover associations between coding variants and related complex traits [18, 19]. In recent years, large-scale exome sequencing studies have provided new insights into the associations between genetic variants and the risk for certain diseases [20–23]. However, it is impossible to fully cover variations with large effects profiled from underrepresented populations by investigations with large-scale data of well-studied populations. These variations, which are associated with biomedical traits, could be influenced by local demographic histories and adaptive processes, some of which benefit from the conservation of isolated circumstances [24–26]. Due to the isolation and the small size of the population, however, little is known about the genetic history and adaptive evolution of most Yunnan minorities. Investigation of these issues could shed light on: (i) the effects of functional variations in protein-coding genes and (ii) the evolutionary adaptations that have shaped the genomes of Yunnan minorities.

To elucidate the population structure, demographic history, and adaptive evolution of Yunnan ethnic minorities in detail, in the present study, we sequenced exomes of four minorities living in Western Yunnan (M.Yunnan.West), including Achang (ACH), Dai (DAI), Deang (DEA), and Jingpo (JIP). These minorities live at different altitudes in the same region: JIP live on hilltops above 1500 m; ACH and DEA live on hillsides at about 1000–1500 m, and DAI live in lowlands below 1000 m [27] (Additional file 1: Table S2 and Additional file 3: Table S1). Based on the tri-genealogy hypothesis, these populations belong to three lineages with distinctive cultures and histories (Additional file 2): ACH and JIP belong to Di-Qiang, DAI belong to Bai-Yue, and DEA belong to Bai-Pu [1]. To the best of our knowledge, we have conducted the first comprehensive and systematic analyses to explore the population history and evolutionary adaptation of these minorities. Moreover, we discuss the rationality of the tri-genealogy hypothesis based on our genetic evidence.

## Results

### Differentiated genetic affinities of Yunnan minorities

Principal component analysis (PCA) in a global context showed that M.Yunnan.West was located between clusters of East Asians and Southeast Asians (Additional file 1: Fig. S3). After removing other worldwide populations while retaining the East Asians and Southeast Asians, the PCA results showed that M.Yunnan.West was not clustered together (Additional file 1: Fig. S3). To further understand the fine-scale genetic affinities of M.Yunnan.West, we selected Han Chinese (HAN) from both South and North China, minorities in South China (M.South), highlander minorities in China (M.Highland), and mainland Southeast Asians (MSEA) as the surrounding populations of M.Yunnan.West (Fig. 1a), and then compared their relationships. As a result, M.Yunnan.West was separated into three distinct clusters in this panel (Fig. 1b and Additional file 1: Fig. S4a): ACH and JIP were clustered closer to the Tibetan (TBN) and other M.Highland, DEA was clustered with the Burmese, and DAI was clustered with the Lahu and Vietnamese. The pattern of the three clusters remained the same when only comparing M.Yunnan.West (Additional file 1: Fig. S4b). We also performed PCA of M.Yunnan.West and their related populations. The results suggest that the different M.Yunnan.West can be distinguished with their related populations in the PC plot (Additional file 1: Fig. S5). The DAI was widely scattered compared with the Vietnamese, probably because of the substructure of DAI in Western Yunnan in our dataset and Southern Yunnan in the Human Genome Diversity Project (HGDP) [28, 29] dataset. We confirmed this substructure by comparing DAI from Western Yunnan in our study and from Southern Yunnan in the 1000 Genomes Project (KGP) [30] and HGDP datasets (Additional file 1: Fig. S6). Overall, these results indicate the genetic components of M.Yunnan.West are differentiated, despite living in the same regions.

We also used the unbiased fixation index (*F*_*ST*_) (Additional file 1: Fig. S7) and the outgroup *f*_*3*_ statistics (Additional file 1: Fig. S8) to examine the genetic relationship of M.Yunnan.West and surrounding populations. The overall genetic makeup of JIP was closest to that of ACH (*F*_*ST*_ = 0.006), followed by surrounding Tibeto-Burman populations such as Burmese, Yi, and Naxi (*F*_*ST*_ = 0.008–0.009). Similarly, ACH was closest to JIP, followed by Tibeto-Burman populations, including Burmese, Yi, and Tujia (*F*_*ST*_ = 0.009–0.01). In contrast, the genetic makeup of DAI was closer to those of MSEA and M.South, such as Vietnamese (*F*_*ST*_ = 0.002), Tujia (*F*_*ST*_ = 0.005), and Cambodian (*F*_*ST*_ = 0.006). DEA had close affinities with surrounding populations such as Burmese (*F*_*ST*_ = 0.007), DAI (*F*_*ST*_ = 0.009), and JIP (*F*_*ST*_ = 0.01). The phylogenetic tree constructed based on the pairwise *F*_*ST*_ also portrayed a similar pattern as PCA: ACH and JIP were located in the clade of M.Highland, and DAI and DEA were located in the clade of MSEA, while DAI was close to the Vietnamese and DEA was close to the Burmese. The results of our outgroup *f*_*3*_ statistical analysis also confirmed our finding that the overall genetic relationship of M.Yunnan.West from three ancient lineages is different from each other.

### Migration and admixture scenarios

**Fig. 2:**
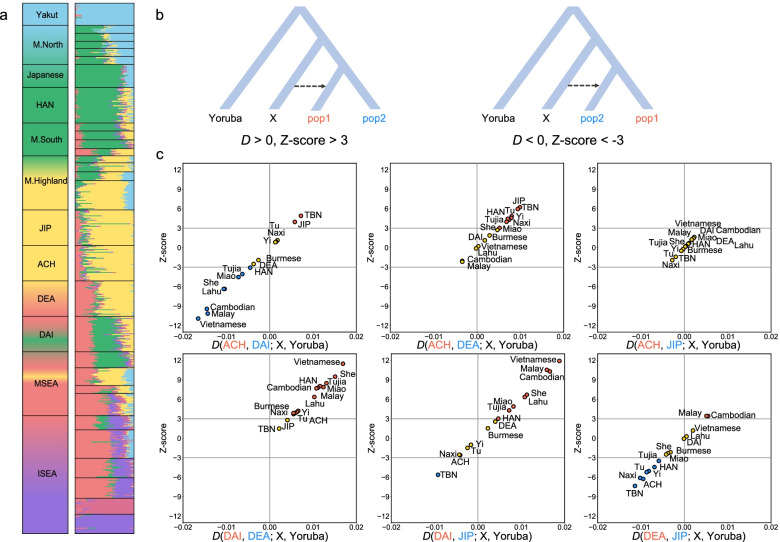
Global ancestry inference and migration signals of M.Yunnan.West. **a** *ADMIXTURE* result of Global Panel B at *K* = 6, using a total number of 28,462 SNPs in 600 samples. Population categories are labeled on the left. ACH, Achang; DAI, Dai; DEA, Deang; JIP, Jingpo; M.North, minorities in North China; M.South, minorities in South China; M.Highland highland minorities in China; MSEA, mainland Southeast Asians; ISEA, island Southeast Asians. **b** The model used for *D* statistic estimations. Positive and negative *D* values indicate the excess allele sharing with population 1 (pop1) labeled in red and with population 2 (pop2) labeled in blue, respectively. An absolute *Z*-score greater than 3 is generally accepted as a strong signal of gene flow. **c** Potential gene introgression of M.Yunnan.West estimated by *D* statistics. Different pairwise M.Yunnan.West population combinations were used as possible populations (pop1 and pop2) under the gene introgression from the assumed ancestor populations in Global Panel C. *D* value above and below 0 for an ancestor population is assumed to have a closer genetic affinity to pop1 and pop2, respectively. An absolute *Z*-score above 3 for an ancestor population is presented in the upper right and lower left, indicating possible gene introgression into pop1 labeled in red and pop2 labeled in blue, respectively

Global ancestry inference with *ADMIXTURE* [31] based on the Global Panel B and C revealed the ancestral makeup of M.Yunnan.West (Fig. 2a and Additional file 1: Fig. S9 and Fig. S10). There were most likely six ancestral populations (*K* = 6) for M.Yunnan.West in Global Panel B based on the estimation of the cross-validation (CV) error (Additional file 1: Fig. S11). The result of *ADMIXTURE* under the Global Panel B indicates that ACH and JIP mainly shared their ancestral makeup with M.Highland (ancestral component colored as yellow, 71.66% ± 15.58% and 75.18% ± 13.94%, respectively), DEA shared the majority of ancestral makeup with M.Highland (ancestral component colored as yellow, 55.01% ± 9.06%) and Southeast Asians (ancestral component colored as red, 30.15% ± 5.69%), and DAI mainly shared their ancestral makeup with lowland East Asians (ancestral component colored as green, 41.53% ± 6.74%) and Southeast Asians (ancestral component colored as red, 29.41% ± 4.06%), suggesting the different genetic origins and admixture histories of M.Yunnan.West populations. In addition, the DEA-specific component was observed at *K* = 9, and the specific component of ACH and JIP was observed at *K* = 10 and further split into two components when *K* = 12 (Additional file 1: Fig. S9), indicating these three populations are more isolated compared to DAI. Similar results were supported by the *ADMIXTURE* inference under the Global Panel C (Additional file 1: Fig. S10). These findings were also confirmed by the estimation of the run of homozygosity (ROH) (Additional file 1: Fig. S12) and identity-by-descent (IBD) sharing (Additional file 1: Fig. S13) under the NGS Panel, illustrating that ACH, DEA, and JIP have a greater number of long ROHs and more IBD segments within populations compared to DAI. One possible explanation could be that more admixture events occurred in DAI due to living in the lowlands with other populations.

Furthermore, we found that the proportion of M.Highland ancestry was correlated with the living altitude of populations under both the Global Panel B and Global Panel C (*R* = 0.85 and *P* = 1.65 × 10^−5^, and *R* = 0.75 and *P* = 5.57 × 10^−4^, respectively) (Additional file 1: Fig. S14a). We also found the M.Highland ancestry was correlated with the longitude of population settlement (*R* =  − 0.56 and *P* = 1.82 × 10^−2^ for Global Panel B, and *R* = 0.74 and *P* = 6.55 × 10^−4^ for Global Panel C), instead of the latitude (*R* = 0.5 and *P* = 4.23 × 10^−2^ for Global Panel B, and *R* = 0.2 and *P* = 0.45 for Global Panel C) (Additional file 1: Fig. S14a). These results indicate that populations living in Western China at higher altitudes are more likely to have a higher ancestral component of M.Highland. To test the major contribution of geographical factors, we further performed an analysis of partial correlations for altitude and longitude by using one as a variable and controlling another one as covariable. We found the significance of altitude (*P* = 2.3 × 10^−2^ for Global Panel B and *P* = 2.71 × 10^−2^ for Global Panel C) was higher than that of latitude (*P* = 0.23 for Global Panel B and *P* = 4.49 × 10^−2^ for Global Panel C) (Additional file 1: Fig. S14b), suggesting the higher contribution of altitude in M.Highland ancestry. In addition, the M.Highland ancestry and Southeast Asian (SEA) ancestry also showed differences among language groups of M.Yunnan.West under both the Global Panel B (Welch’s ANOVA *P* = 7.04 × 10^−52^ and *P* = 8.76 × 10^−46^, respectively) or C (Welch’s ANOVA *P* = 7.31 × 10^−29^ and *P* = 1.02 × 10^−40^, respectively) (Additional file 1: Fig. S14c). A similar result was obtained when surrounding populations were added to the corresponding language groups (Additional file 1: Fig. S14d). Overall, these findings indicate that the different living altitudes and languages could be the causes of the distinctive admixture patterns, which further lead to the genetic differences among M.Yunnan.West.

To further infer the potential admixture of M.Yunnan.West, we applied the *D* statistics to detect the gene flow signals. In the *D* statistic models, positive *D* values indicate more allele sharing with the first population and negative *D* values indicate more shared alleles with the second population; absolute *Z*-scores greater than 3 are generally accepted as a strong signal of gene flow (Fig. 2b). As a result, a significant gene flow signal from TBN was detected in ACH and JIP, followed by other M.Highland, possibly illustrating the migration from populations living in the Tibetan area to ACH and JIP. Besides, DAI showed multiple signals of gene flow from surrounding populations, including MSEA, M.South, and HAN, suggesting that frequent admixture events occurred in DAI (Fig. 2c). In contrast, few signals of gene flow were detected in DEA, which proves DEA is the most isolated population among M.Yunnan.West (Fig. 2c). In addition, we found no gene flow signal when the two target populations were ACH and JIP due to the high similarity of their genetics (Fig. 2c). However, the negative *D* values of M.Highland indicated JIP has a slightly closer genetic affinity with highlander minorities than ACH, which is consistent with the PCA results. We conducted the same analysis based on the *f*_*3*_ statistics and obtained similar results (Additional file 1: Fig. S15).

At last, we applied *dadi* [32] to analyze the migrations of M.Yunnan.West using the data of the NGS Panel. Population joint site frequency spectrum (SFS) based on putatively neutral sites was used to infer population migration history (Additional file 1: Fig. S16 and Fig. S17). We estimated the pairwise migration rate and direction among populations using the symmetrical migration model (SMM) and asymmetrical migration model (AMM) (Additional file 1: Fig. S16a). Based on the results of the SMM (Additional file 1: Fig. S16b and Additional file 4: Table S3), we found that ACH had a high migration rate with JIP, and JIP had a high migration rate with TBN. Further, the DAI had a high migration rate with HAN, and DEA had a high migration rate with M.Yunnan.West in the same region, including AHC and DAI. We also found that the populations living at higher altitudes were less likely to have migration events with HAN. To further detect the migration direction, we applied AMM (Additional file 1: Fig. S16c and Additional file 4: Table S3). The ratio of migration rate between TBN and M.Yunnan.West revealed that the direction of migration events is from TBN to M.Yunnan.West. In addition, the migration rates of DAI and HAN were approximately equal, indicating the unbiased migration between these two populations. These results were supported by the analyses conducted by *TreeMix* [33] (Additional file 1: Fig. S18).

### Demographic history of Yunnan minorities

**Fig. 3:**
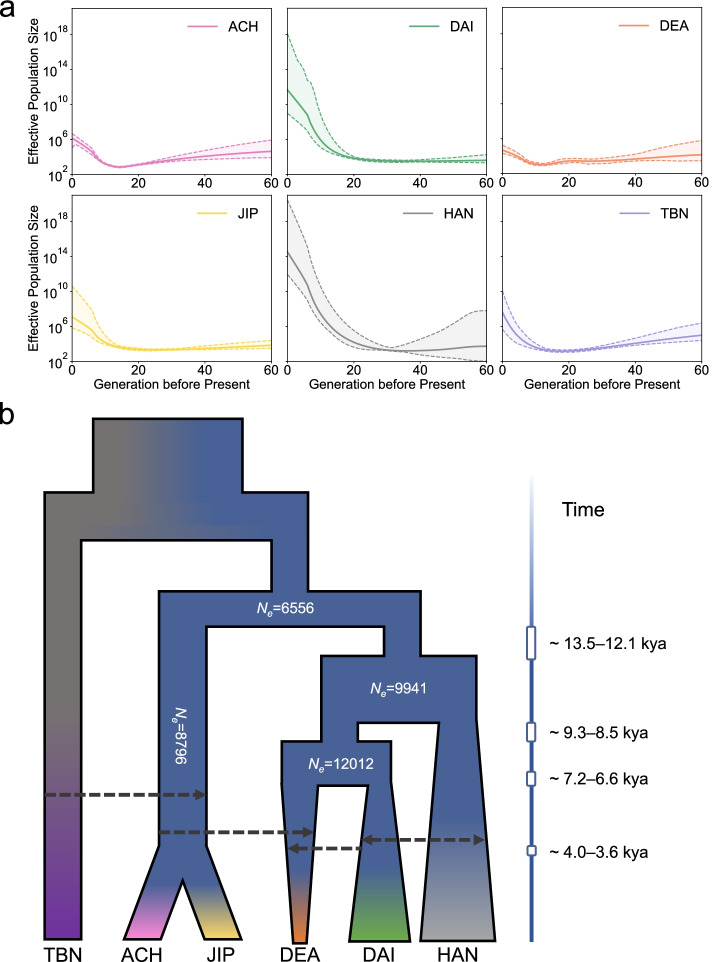
Recent effective population size and integrated demography model of M.Yunnan.West. **a** Recent demographic histories inferred by *IBDNe*, using populations from the NGS Panel. **b** Integrated demography model describing the population history of M.Yunnan.West. Population divergence and effective population size were estimated by the 5-population model of *dadi*. Detected population migrations based on the results of *D* statistics, *dadi*, and *TreeMix* are portrayed as bold dotted arrows

Data from the NGS Panel were used to infer demographic population history. We utilized *IBDNe* [34] to infer the recent demographic history of M.Yunnan.West (Fig. 3a). We found that populations who have an M.Highland component of > 50%, including ACH, DEA, JIP, and TBN, all showed a bottleneck around 15 generations ago. Besides, HAN and DAI depicted larger effective population sizes (*N*_*e*_) than other populations, which is consistent with previous studies reporting large *N*_*e*_ values for HAN and DAI among East Asians [35, 36]. We also estimated the *N*_*e*_ using the LD-decay approach (Additional file 1: Fig. S19a), which allows us to infer *N*_*e*_ more than 200 generations ago. The results based on the LD-decay method also showed that the *N*_*e*_ values of HAN and DAI were consistently larger than those of other populations, indicating the consistent expansion of these two populations.

We further estimated the pairwise divergence time using *dadi* (Additional file 1: Fig. S16d and Additional file 4: Table S3) based on the comparison of the expected and observed SFS (Additional file 1: Fig. S17). We applied different demography models and calculated the log-likelihood for each model to determine the best-fit divergence time. As a result, we inferred that ACH and JIP had the latest divergence time (3900 ya, 95% confidence interval [CI]: 3400–4200 ya), and JIP had a later divergence time with TBN (6700 ya, 95% CI: 6000–7100 ya) than other populations. The divergence between DAI and the M.Highland ancestry-enriched populations, ACH and JIP, was 7000 ya (95% CI: 6400–8200 ya) and 6900 ya (95% CI: 5500–7400 ya), respectively, which is earlier than that of DEA and HAN. The divergences of DEA from populations in the same region, ACH, DAI, and JIP, are 7100 ya (95% CI: 6200–8700 ya), 5500 ya (95% CI: 4200–6600 ya), and 6400 ya (95% CI: 5700–7500 ya), respectively, being more recent than those from other populations. The estimated divergence time was also consistent with the results inferred by the LD-decay method (Additional file 1: Fig. S19b). Although the values estimated by the LD-decay method were lower than those calculated with *dadi*, the overall relationships among the populations were consistent with those suggested by *dadi*.

After modeling with pairwise 2-population models, we hypothesized a model topology based on the pairwise best-fit divergence time inferred from 2-population models in *dadi*, and utilized a hypothesis-testing framework of 3-population models (see the “Methods” section) to confirm this topology was the best-fit one of the four M.Yunnan.West populations and two reference populations (Additional file 5: Table S4) (see the “Methods” section). The model topology was also recovered by the inferred maximum likelihood tree of the *TreeMix* (Additional file 1: Fig. S18). After the confirmation of model topology, we estimated the *N*_*e*_ and population divergence of the five populations except the outgroup TBN using the 5-population model in *dadi* (Additional file 1: Table S5). Taken together, we propose an integrated model to describe the population history of M.Yunnan.West (Fig. 3b). As the model shows, the Tibeto-Burman speaking populations ACH and JIP are affected by the gene flow from the highland minority TBN. The DAI is genetically close to DEA and there is a mutual gene flow between DAI and HAN. The DEA is affected by gene flow from the populations with M.Highland ancestry (ACH and JIP) and populations with SEA ancestry (DAI).

### Novel variants identified in Yunnan minorities

Based on the results of ROH and IBD sharing, we assumed that ACH, DAI, DEA, and JIP show differences in genetic diversity and novel variants. Using HAN for comparison, we first calculated the average nucleotide differences ($\overline{\pi }$) of each M.Yunnan.West population and found that DEA showed the highest value, followed by ACH, JIP, and DAI (Additional file 1: Fig. S20a). We then calculated novel variants that were defined as variants not presented in the dbSNP v154 [37] and Exome Aggregation Consortium (ExAC) [38] datasets, at the population level and the individual level. We found that most novel variants were singletons in these populations, especially in the reference population HAN, due to the large sample size (Additional file 1: Fig. S20b). After removing singletons, we observed that ACH, DEA, and JIP harbored a significant proportion of novel variants that were not represented in public databases, and DEA showed the greatest number of novel variants, followed by ACH and JIP (Additional file 1: Fig. S20b). A similar result was obtained at the individual level, irrespective of whether singletons were included (Additional file 1: Fig. S20c).

Among M.Yunnan.West populations, we observed that DEA showed the largest average number of population-specific novel variants no matter removing singletons or not, followed by ACH, JIP, and DAI (Additional file 1: Fig. S21a). To further investigate the variant type of these novel variants, we annotated variant consequences using the Ensembl Variant Effect Predictor (*VEP*) [39] and counted the number of each annotation category (Additional file 1: Table S6). We defined variants with a high impact classification and missense variants whose SIFT [40] and PolyPhen [41] scores in *VEP* both predicted that they are harmful as loss-of-function (LOF) variants (Additional file 1: Table S6 and Additional file 6: Table S7). We then found that while ACH, DEA, and JIP showed a higher number of LOF novel variants than DAI (Additional file 6: Table S7), probably because most novel variants of DAI were reported in the KGP and HGDP datasets, the proportion of LOF variants was highest in DAI (Additional file 1: Fig. S21b), indicating a higher genetic burden in DAI.

### Shared and divergent adaptation in Yunnan minorities

**Fig. 4:**
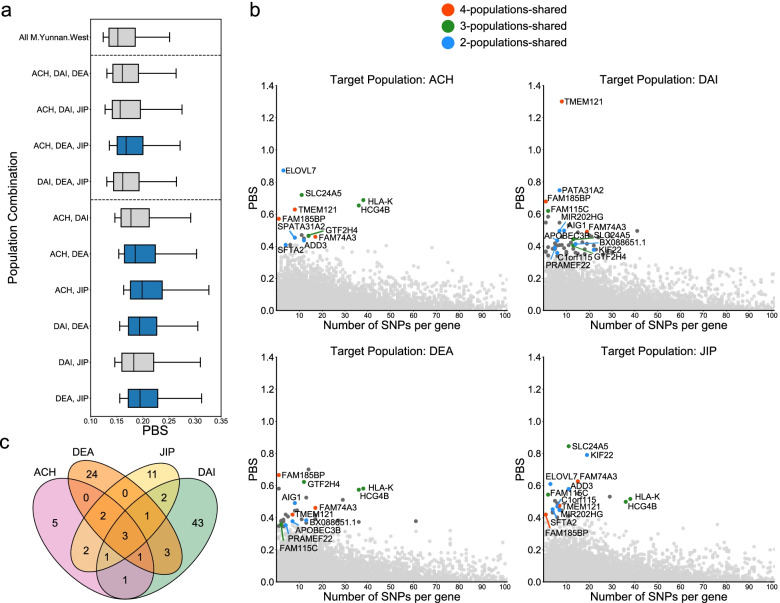
Shared adaptive signals among M.Yunnan.West. **a** PBS distribution of genes over the 99th percentile, using different combinations of M.Yunnan.West populations. Box plot outliers were removed. Blue boxes indicate population combinations sharing the apparent same ancestral makeup. **b** Shared adaptive signals with extreme significance (simulation *P* value less than 0.01) among M.Yunnan.West populations. Only signals shared by at least two populations are labeled. **c** Venn plot representing the overlaps of extremely significant shared adaptive genes in four M.Yunnan.West populations

To detect the shared signals of adaptive evolution among M.Yunnan.West populations, we calculated the Population Branch Statistic (PBS) for each gene [42], using HAN and CEU as the second and third populations, respectively. This allowed the detection of genes that are likely under selection in all of the M.Yunnan.West populations but not in HAN. To estimate the significance of PBS values, we simulated PBS values under the demographic model inferred from this study using CEU as an outgroup and compared the generated data with observed data. We combined different M.Yunnan.West populations into one population and calculated the PBS values over the 99th percentile, and then we compared the distributions of different population combinations (Fig. 4a). We found that the populations that shared a higher ancestral component also showed higher PBS values. For example, a combination of M.Highland ancestry containing ACH, DEA, and JIP showed a higher PBS value than other three-population combinations. This indicates that M.Yunnan.West populations that share a higher ancestral component tend to share more adaptive signals. We also calculated the PBS values of genes for each population and selected genes with a *P* value below 0.01 as the extreme adaptive signals (Fig. 4b). DAI showed a greater number of extremely significant signals than the other three populations (Fig. 4c), which suggests that the number of genes that are likely under selection in DAI but not in HAN is higher than for the other three populations. Moreover, we found that *FAM185BP*, *FAM74A3*, and *TMEM121* were extremely significant selection signals for all M.Yunnan.West populations. *TMEM121* expression levels are often determined in skin biopsies, and *TMEM121* has been reported to play a role in endothelial cells and chronic inflammatory disorders [43]. In addition, two genes related to the major histocompatibility complex (MHC) in humans, *HLA-K* and *HCG4B*, were identified as a significant signal in ACH, DEA, and JIP. The gene *SLC24A5*, which is involved in skin pigmentation, also showed a significant signal in ACH, DAI, and JIP. Other genes shared between the three populations were *GTF2H4* in ACH, DAI, and DEA and *FAM115C* in DAI, DEA, and JIP.

**Fig. 5:**
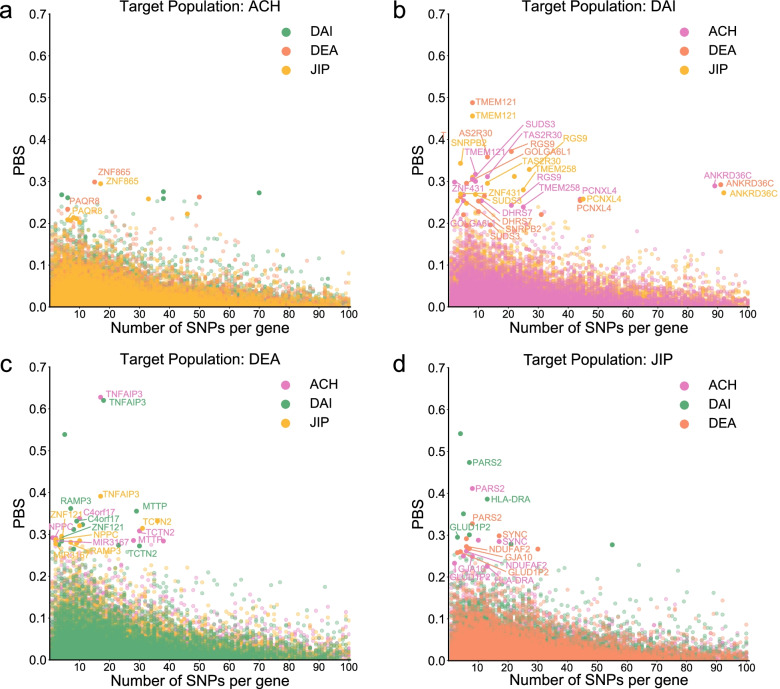
Differential adaptive signals among M.Yunnan.West. Differential adaptive genes with extreme significance (*P* value less than 0.01) were scanned by PBS in **a** ACH, **b** DAI, **c** DEA, and **d** JIP. Different colors indicate that M.Yunnan.West populations were used as the second population and HAN was used as the third population in each calculation. Only signals detected as differential genes in at least two populations are labeled

To investigate genes under selection specific to one M.Yunnan.West population, we calculated PBS values using each non-target M.Yunnan.West population as the second population and HAN as the third population [25]. Following the same process above, genes with a *P* value less than 0.01 were selected as extreme adaptive genes for each M.Yunnan.West populations (Fig. 5). Most of these genes also showed evident signatures of natural selection as estimated by cross-population extended haplotype homozygosity (XP-EHH), using HAN as the reference population (Additional file 1: Fig. S22).

We further identified and annotated variants with PBS values greater than 0.1 as major contributing variants for these differential adaptive genes (Additional file 7: Table S8). Regarding DAI, a population that prefers bitterness as a special eating habit, we identified the gene *TAS2R30*, which plays a role in the perception of the bitter taste [44, 45]. Derived allele frequencies (DAFs) of missense variants for *TAS2R30* were all 0% in DAI but 10%–40% in other M.Yunnan.West populations. Other genes with major contributing missense variants in DAI included *ANKRD36C*, involved in spermatogenesis, *PCNXL4*, and *GOLGA6L1*, involved in immunity, and *TMEM121*, which plays a role in human skin biopsies. In addition, we identified *C4orf17* genes in DEA. This gene is highly related to alcohol metabolism. *MTTP*, which is involved in lipid metabolism, is affected by alcohol exposure [46] and was reported to be associated with alcoholic fatty liver in the Korean population [47]. The remaining genes of DEA with major contributing missense variants were *RAMP3*, involved in the regulation of the vascular system, and *TNFAIP3*, involved in immunity. In JIP, we identified the *SYNC* gene, which is involved in the formation of muscle fiber and consistently shows a high expression level in muscle tissue in the Genotype-Tissue Expression (GTEx) v8 dataset [48] (Additional file 1: Fig. S23). Other genes with contributing missense variants in JIP were *PARS2*, associated with mitochondrial disease [49], and *HLA-DRA*, which plays a key role in the human immune system.

To confirm the divergent adaptation of M.Yunnan.West populations is related to biomedical pathways, we took the intersection of genes with a strong adaptive signal (*P* < 0.05) in all three comparisons for each target M.Yunnan.West population as the gene sets for enrichment analysis (Additional file 8: Table S9). Functional enrichment and protein − protein interaction (PPI) network analyses were performed using *metascape* [50]. If − log_10_(*P* value) > 2, a functional category was considered significant. As a result, 11 functional groups were recognized for ACH, 20 for DAI, 19 for DEA, and 11 for JIP (Additional file 1: Fig. S24 and Additional file 9: Table S10); 2 PPIs were identified for ACH, 4 for DAI, 2 for DEA, and 1 for JIP (Additional file 1: Fig. S25). Strikingly, some of these functional categories are possibly associated with the habits and adaptations to living circumstances of different M.Yunnan.West populations, such as categories associated with mitochondrial alteration (GO: 0070125 and GO: 0010821) in ACH, categories associated with malaria (KEGG: hsa05144), glucose metabolism (GO: 0042149 and GO: 0010907), and taste transduction (KEGG: hsa04742) in DAI, and a pathway related to alcohol (GO: 0097305 and GO: 0097306) in DEA.

## Discussion

### Genetic evidence of the tri-genealogy hypothesis

In this study, we unraveled the genetic components of M.Yunnan.West populations and found they are distinctly differentiated and genetically divided into three groups. Living in the same region, the three groups live at different altitudes and speak different languages. Our observations confirmed that these three groups have distinctive genetic patterns. We propose that the complex landforms and different language families could be the reason behind the lack of genetic exchange and the maintenance of persistent genetic differentiation (Additional file 1: Fig. S14). These differentiations could be attributed to the different migration and regional adaptations along with the population history and have been further validated in our study. Our observation, to some extent, is in agreement with the tri-genealogy hypothesis, which is mainly based on linguistic studies. ACH and JIP belong to the Di-Qiang lineage, showing a genetically close affinity with highland minorities speaking the Tibeto-Burman language. The DAI belongs to the Bai-Yue lineage speaking the Tai-Kadai language and had close genetic affinities with MSEA and lowland East Asians. DEA belongs to the Bai-Pu lineage and an Austroasiatic language, appearing to be related to both M.Highland and MSEA. This tri-genealogy hypothesis has been widely used in studies of Yunnan minorities and in some ways makes sense since it is generally assumed that populations in the same language families tend to show similar genetic patterns because of migration and genetic drift [51].

However, this hypothesis is based on ethnic and linguistic records, and many limitations were shown in the context of the rapid development of human genetics. Previous studies analyzed the NRY haplogroup of Yunnan minorities of these three ancient lineages and found that they have certain typical characteristics but could not be clearly distinguished from each other [2]. The Lahu is an ethnic minority speaking a Tibeto-Burman language and is thus classified as belonging to the Di-Qiang lineage, but in the present study, we found that the Lahu showed little genetic affinity with the highland minorities, and similar to the DAI, the Lahu were more related to M.South and Southeast Asians (Additional file 1: Fig. S9). One possible reason for this discrepancy could be that the Lahu do not live in Northwestern Yunnan like most other Di-Qiang minorities, but in Southwestern Yunnan, which is dominated by minorities of Bai-Yue and Bai-Pu lineages. Languages can change easily when a small population is merged with a large one [2]. We propose that the formation of different Yunnan minorities is influenced by many other factors, such as geographical location, migration, and admixture history, besides the language family, making the tri-genealogy hypothesis outdated in some cases. More studies are also needed to further investigate the complex population history and genetic affinity of the diverse ethnic minorities in Yunnan.

### Distinct demographic history of Yunnan minorities

Based on the historical records, ACH and JIP are descendants of the ancient Di-Qiang. They migrated from Southeastern Tibet along the Tibetan-Yi corridor and the ancient Tea-Horse Road [8, 51]. In the present study, we observed ACH and JIP shared a high ancestral component with M.Highland from the result of *ADMIXTURE* (Fig. 2a). In addition, a previous study using Y-chromosomal data found that JIP harbors a high frequency of the NRY haplogroup D-M174, which is prevalent in M.Highland [17]. These results suggest that ACH and JIP are originated from the Tibeto-Burman speaking groups living in the highlands. We also detected the gene flow from M.Highland led by the TBN to ACH and JIP, using the *D* statistics (Fig. 2c), *dadi* (Additional file 1: Fig. S16), and *TreeMix* (Additional file 1: Fig. S18). Our observations confirmed that the migration from the Tibetan area to Western Yunnan resulted in the present-day ACH and JIP populations. However, ACH, DEA, and JIP that contained the M.Highland ancestry of > 50% all show a recent bottleneck around 15 generations (Fig. 3a). One explanation for the bottleneck could be the chaos caused by the drastic struggle among competing Buddhists in the Tibetan area around the sixteenth century in the Ming dynasty [52].

The Bai-Yue populations are widely distributed in South China and mainland Southeast Asia, and the DAI belonging to the Bai-Yue populations has been used as one of the representative minorities of China in public human genome datasets such as KGP [30] and HGDP [28]. In the present study, we found that the DAI shared a higher proportion of ancestral components with M.South and MSEA compared with other M.Yunnan.West, suggesting the DAI is originated from the ancient Bai-Yue populations. The previous studies showed that DAI has the larger *N*_*e*_ value among East Asians [35, 36]. Our observations based on *IBDNe* suggest that DAI experienced a different demographic history than the other three M.Yunnan.West populations, which connotes that DAI did not experience population bottlenecks in recent generations (Fig. 3a). In addition, based on the *ADMIXTURE* (Fig. 2a, Additional file 1: Fig. S9, and Additional file 1: Fig. S10), ROH (Additional file 1: Fig. S12), and IBD sharing (Additional file 1: Fig. S13) results, we found that DAI was less isolated than ACH, DEA, and JIP. The results of *D* statistical analyses also indicate that DAI received more gene flow than the other three M.Yunnan.West populations, mainly from MSEA, M.South, and HAN (Fig. 2c). Since the implementation of the Tusi System in the Yuan Dynasty, most minorities in ancient Southwest China were under the control of DAI, resulting in the DAI having a higher social status among these southwestern minorities. This may also be explained by our observations that the DAI is more frequently admixed with surrounding populations and undergo continuous population expansion.

As an ethnic minority belonging to the Bai-Pu lineage that was initially called the Ailao tribe, the DEA is recognized as an indigenous population living in Western Yunnan. Our observation based on the *ADMIXTURE* and *D* statistics suggests that the DEA was the most isolated one of the M.Yunnan.West populations, suggesting the DEA has a different genetic origin from surrounding population groups. In addition, the different genetic origins with little expansion and migration in DEA possibly resulted in the high number of novel variants compared with other M.Yunnan.West populations. With the development of the Ailao tribe of Bai-Pu lineage, the Ailao state was gradually established under the rule of DAI expanding from the South. In addition, the Di-Qiang lineage from the North also reached the Ailao state to escape the war. As a result, the Bai-Pu lineage was probably influenced by both Di-Qiang and Bai-Yue lineages during history. Our results confirm that DEA shares the ancestral components with M.Highland and MSEA, which are the representative ancestral components of the Di-Qiang and Bai-Yue lineages, respectively.

### Local adaptation of M.Yunnan.West populations

We first searched for shared signatures of selection in M.Yunnan.West populations by scanning genes using PBS (Fig. 4c). We identified three genes with extreme significance, i.e., *FAM185BP*, *FAM74A3*, and *TMEM121*. *FAM185BP* and *FAM74A3* are long non-coding RNA genes that have not been fully characterized. *TMEM121* is used in skin biopsies and plays a role in endothelial cells and chronic inflammatory disorders [43]. Another study also indicated that *TMEM121* plays a role in immunity related to skin diseases such as psoriasis [53]. With respect to extremely significant adaptive signals shared in three populations, we identified *HLA-K* and *HCG4B* in ACH, DEA, and JIP, *SLC24A5* in ACH, DAI, and JIP, *GTF2H4* in ACH, DAI, and DEA, and *FAM115C* in DAI, DEA, and JIP. Among these genes, *HLA-K*, *HCG4B*, and *GTF2H4*, belonging to the MHC gene family, and *SLC24A5*, involved in skin pigmentation, were previously reported as candidate genes favored by natural selection in human populations [54, 55]. In addition, most of these genes, including *HLA-K*, *HCG4B*, *GTF2H4*, and *FAM115C*, play key roles in the regulation of the human immune system. For example, *FAM115C* is related to cancer cell migration [56, 57], and *GTF2H4* plays a key role in DNA repair and has been reported to be associated with the risk for human papillomavirus (HPV) resistance in Costa Ricans [58]. Concerning the divergent adaptations, the DAI showed more differential adaptive variants than other M.Yunnan.West populations (Figs. 4c and 5). We identified the differential genes specific to each M.Yunnan.West population (Additional file 7: Table S8). Furthermore, we performed enrichment analysis for differential genes specific to each M.Yunnan.West population, using the intersection of adaptive variants strongly differentiated from each of the other three M.Yunnan.West populations (Additional file 8: Table S9). Notably, results of differential adaptive genes and functional enrichment categories illustrated specific adaptive evolution possibly related to the living environment and habits of each M.Yunnan.West population (Additional file 9: Table S10).

As a multifactorial disorder, hypertension has been reported to be associated with mitochondrial alterations [59]. In our study, we profiled *PARS2*, which is associated with mitochondrial disease, as an extremely differential gene in JIP. The functional category of metabolic disorders of biological oxidation enzymes (R-HSA-5579029) was also enriched in the JIP differential gene set. Additionally, functional categories related to mitochondrial alterations, including mitochondrial translational elongation (GO: 0070125) and regulation of mitochondrion organization (GO: 0010821), were enriched in the differential gene set of ACH. The ancient Di-Qiang populations were living in areas with high altitudes like Tibetan areas. The habit of living in the high altitude of the local environment was kept in ACH and JIP after they migrated to Western Yunnan, while such continuity of residence was lacking in the ancestors of DAI and DEA. A previous study indicated that hypertension is more prevalent in Yunnan minorities of Di-Qiang lineages, such as Bai and Tibetan [60]. Therefore, we speculate that the differences in living habits contribute to the detection of hypertension-related selection signatures within M.Yunnan.West. These results suggest that ACH and JIP, as descendants of the Di-Qiang lineage living in the highlands, are likely under adaptive evolution of hypertension.

The ancestor of DAI is one of the indigenous populations living in South China in the past. The present-day DAI people mainly live in the lowlands of Southwestern Yunnan, which is permanently hot and humid and where malaria was prevalent in the past [61]. These special living circumstances give rise to the specific adaptive evolution of DAI. On the one hand, the hot and humid environment drives the DAI to prefer bitterness as their eating habit because the ancient DAI believed that bitterness could dispel dampness and detoxify the body. Among the extremely significant differential adaptive genes for DAI, *TAS2R30* is highly associated with the perception of bitter taste. The functional pathway of taste transduction (KEGG: hsa04742) was also enriched in the DAI gene set of strong differential adaptive signals. A previous study indicated that the genetic diversity of the human *TAS2R* gene family is higher than the genome-wide average due to the elevated rates of non-synonymous substitution [62]. In the present study, most of the major contributing variants identified in *TAS2R30* were non-synonymous, and DAFs of these missense variants were all 0% in DAI but 10–40% in other M.Yunnan.West populations. With regard to other M.Yunnan.West that have no traditional habit of eating bitter foods, sensitive bitterness perception would safeguard them from eating toxic substances [63]. Conversely, the *TAS2R* under selection in DAI may be explained by their low sensitivity to the bitter taste.

Malaria in Yunnan has been documented since 225 AD. It has been a persistent epidemic disease in Yunnan until the 1950s [64]. The border and low-altitude areas of Yunnan are the main malaria-endemic areas [65] and highly overlap with the living areas of DAI. In this study, we identified genes related to the malaria resistance pathway (KEGG: hsa05144), including *CCL2*, *CD40*, *HBA1*, and *HBA2* as the differential genes in DAI, indicating different selective pressure posed by malaria on the DAI compared to other M.Yunnan.West. In addition, we found that *HBA1* and *HBA2*, genes involved in malaria resistance by the sickle cell and thalassemia traits [66–68], which also explains the high prevalence and specific pattern of thalassemia in the present-day DAI [69, 70]. Similarly, glucose-6-phosphate dehydrogenase (G6PD) deficiency is one of the common enzymopathies affecting people living in regions where malaria is endemic, as a result of natural selection against genes that are associated with susceptibility to malaria [71–73]. In our results, functional categories related to the response to glucose, including cellular response to glucose starvation (GO: 004214) and positive regulation of glucose metabolic process (GO: 0010907), were enriched in the differential gene set of DAI, which could be a consequence of G6PD deficiency. The G6PD deficiency can cause the blockade of the pentose phosphate pathway and the accumulation of its substrate, glucose-6-phosphate, to compensate for the glucose metabolic process and further reduce the sensitivity of cells to glucose starvation [74]. At the same time, the G6PD deficiency can lead to a decrease in the production of NADPH, which will increase the level of oxidation of erythrocyte and further result in hemolytic anemia [75, 76]. Thus, these enriched terms from the differential gene set of DAI suggest the DAI might be under the adaptive process to the G6PD deficiency due to the malaria prevalence. Although ACH, DEA, and JIP have also been reported to suffer from malaria, their susceptibility and severity of malaria were lower than that of the DAI since they live in high-altitude areas with a relatively cold and dry environment. Accordingly, the onset and prevalence of thalassemia and G6PD deficiency in these populations could also be different from that of the DAI [70, 77, 78].

As one of the genes involved in divergent adaptation in DEA, *C4orf17* is the nearest flanking gene of *ADH*s, which are the well-known genes related to alcohol metabolism. Previous large-scale genome-wide association studies revealed that *C4orf17* is significantly associated with alcohol consumption at the gene level [79]. Moreover, another differential gene, *MTTP*, which encodes a triglyceride transporter, showed a strong signature of selection in Southeast Asians in a previous study [80]. As the near downstream gene of *C4orf17*, *MTTP* has been suggested to be related to alcoholic fatty liver disease [47], possibly because alcohol exposure could increase triglyceride and cholesterol levels [46]. In addition, functional categories involved in the response to alcohol (GO: 0097305 and GO: 0097306) were also enriched from the differential gene set of DEA. The ACH, JIP, and DAI are all considered to be the populations with prosperous liquor cultures, while the ancient DEA had neither winemaking technologies nor liquor culture. This indicates that DEA was less able to undergo the adaptive process of the improvement of alcohol consumption, which possibly explains that selection signals related to alcohol metabolism were differentiated in DEA from the other three M.Yunnan.West populations.

### Limitations of this study

In this study, we analyzed the genetic structure, population history, and local adaptations of 4 minority groups in Western Yunnan, and most of the results were based on the analyses of the target region of whole-exome sequencing (WES) data. Since our studied populations are different genetic backgrounds and WES data were less used in population genetic studies, we incorporated diverse populations from both whole-genome sequencing (WGS) and genotyping array data like references and designed different dataset panels based on the different analysis purposes. For example, we included both sequencing data and genotyping array data in analyses of population structure, but only used sequencing data in analyses of population history and local adaptation due to the ascertainment bias of genotyping array data [81]. Such study design with different datasets is effective in eliminating the potential bias stemming from multiple data resources.

The rare variants and unascertained common variants can be identified from WES data, which enables inferences of demographic history based on the SFS [82]. However, due to the lack of non-coding regions that include a great number of common variants, WES data are not as powerful as the WGS data for inference of population history. For example, applying MSMC [83] to infer long-term *N*_*e*_ was only supported by the WGS data. In addition, genetic inference from WES data may be subject to selective pressures since exons contain a substantial proportion of the functional variants [82]. Although some data processing such as only including evolutionary neutral sites as we used in this study (see the “Methods” section), were used to reduce the effect of background selection in CDS regions [25, 84], the analysis results based on the exome target region could inevitably be biased from the genome-wide level that may be closer to the real population history. Thus, due to the limitations of WES data, there would inevitably be potential biases in our results compared to the genome-wide level.

## Conclusions

In this study, we characterized genetic structures, population histories, and local adaptations of Yunnan minorities. We found that Yunnan minorities from three ancient lineages, i.e., Di-Qiang, Bai-Yue, and Bai-Pu, show sufficient genetic differences. We modeled the population history of Yunnan minorities from three ancient lineages and provide genetic evidence for the tri-genealogy hypothesis. Di-Qiang populations are related to highland minorities and likely migrated from the Tibetan area about 6700 years ago. The divergence time between Bai-Yue and Di-Qiang was estimated to be 7000 years, and that between Bai-Yue and Bai-Pu was estimated to be 5500 years. Bai-Pu is relatively isolated with few expansions, but evidence of gene flow from surrounding Di-Qiang and Bai-Yue populations was also found. Adaptive variants possibly associated with the living circumstances and habits of the Yunnan minorities were identified. A few functional mitochondrial alterations and *TAS2R30* might be associated with a higher incidence of hypertension in Di-Qiang populations. Adaptive variants related to malaria and glucose metabolism were identified in the Dai population and indicate the adaptation to thalassemia and G6PD deficiency resulting from malaria resistance, while selection on *PARS2* is likely related to the perception of bitterness. *C4orf17* and *MTTP* in Deang are associated with alcohol metabolism and the potential adaptation to the alcohol response.

## Methods

### Sample collection, exome sequencing, and SNP-calling

Epstein-Barr virus immortalized B lymphoblastoid cell lines (LCLs) from 242 native ethnic individuals, including 65 Achang (ACH), 52 Dai (DAI), 65 Deang (DEA), and 60 Jingpo (JIP) living in Mangshi of Dehong Autonomous Prefecture, Yunnan Province (Fig. 1a and Additional file 3: Table S1), were obtained from the Immortalize Cell Bank of Chinese Ethnics Groups hosted in the Institute of Medical Biology, CAMS. Whole exome sequencing (WES) data with high target coverage (100 × –150 ×) for 150 bp paired-end reads was carried out on the Illumina Hiseq X10 platform (iGeneTech, Ltd., Beijing, China).

*Trimmomatic* v0.4.0 [85] was applied for raw FASTQ data trimming using the recommended default parameters. Reads of each sample were mapped to the human reference genome (GRCh37) using *BWA-MEM* v0.7.10 [86]. We executed duplicate mark and base quality recalibration using *GATK* v3.8 [87]. Variants calling was performed through the *HaplotypeCaller* module of *GATK* based on the GVCF mode.

For comparison, WES data of 300 Han Chinese (HAN) residing in different regions of China from the HuaBiao Project [88, 89] were also collected (Additional file 1: Table S2). We performed a joint variant calling of M.Yunnan.West with HAN samples as well as 33 whole-genome sequenced (WGS) Tibetan (TBN) samples with high coverage collected from Lu et al. [90, 91] and implemented strict quality control through VQSR. We filtered the raw variant calling file into the target region of 62,984,579 base pairs. As a result, bi-allelic single-nucleotide variants with high quality were retained for downstream analyses.

### Data compilation

To analyze the genetic variation of M.Yunnan.West in a broader context, we collected the global populations from the Human Genome Diversity Project (HGDP) dataset in the WGS version [29, 92]. Since Yunnan province is close to mainland Southeast Asia and there are much fewer Southeast Asians in HGDP dataset, we also collected genotype data including 196 Southeast Asians from Mörseburg et al. [93, 94]. We combined our joint-calling dataset, HGDP dataset, and 196 Southeast Asians as the Global Panel. There are 46,845 SNPs retained in this dataset after filtering SNPs with a missing rate higher than 10%. To solve specific problems under different contexts, we dissect Global Panel into different subsets. Global Panel A contained the whole global population of the Global Panel, Global Panel B contained the East Asian and Southeast Asian included in Global Panel, and Global Panel C only included M.Yunnan.West and their surrounding populations (M.South, M.Highland, MSEA, and HAN) (Additional file 3: Table S1). Besides, we also collected the altitude information of populations in Global Panel C based on the information of longitude and altitude using Google Earth (https://earth.google.com) (Additional file 1: Table S2). The maps used in this study, including GS(2016)1609, GS(2016)1667, and GS(2020)4618, were obtained from a standard map service (http://bzdt.ch.mnr.gov.cn) approved by the Ministry of National Resources of the People’s Republic of China.

The Global Panel shows limitations for insufficient genetic information due to the lack of joint-calling and the merging of genotype data. To conduct more comprehensive analyses, including estimating effective population size (*N*_*e*_), population divergence time, and PBS calculation, we used our joint-calling dataset as NGS Panel. This panel is informative and contains 274,634 SNPs, which enables analyses requiring sequencing data input.

### Population structure and genetic affinity

To avoid the bias caused by a close genetic relationship, we identified the relatedness of M.Yunnan.West samples using the *KING* v2.1.2 [95] and excluded samples within the second-degree relationship for subsequent population structure analyses (Additional file 1: Fig. S2). We used the dataset from Global Panel and performed a series of principal component analyses (PCA) at the individual level using the *SNPRelate* v1.16.0 [96]. We restricted every single dataset in the target region and selected bi-allelic autosomal SNPs with a missing rate of less than 0.05 using the *BCFTools* v1.6 [97]. *PLINK* v1.9 [98] was applied to perform LD-pruning of SNPs using a window of 1,000 base pairs advanced by 100 SNPs at a time and an r-squared coefficient of 0.2 for the combined dataset. After quality control, 17,101 SNPs were left for subsequent analyses.

Global ancestry inference under the Global Panel B and Global Panel C was performed by *ADMIXTURE* v1.3.0 [31] to dissect the ancestral makeup of each individual. The input data for admixture analysis were prepared using the same method as for the PCA. To avoid the bias caused by differences in sample sizes, we randomly selected 40 samples for populations with larger sample sizes.

Population differentiation was estimated following Weir and Cockerham’s *F*_*ST*_ [99] using *SNPRelate* v1.16.0. To investigate the relationship between M.Yunnan.West and their surrounding populations, we calculated pairwise *F*_*ST*_ values among populations in Global Panel C and constructed a phylogenetic tree based on the pairwise *F*_*ST*_ results.

We also applied the outgroup *f*_*3*_ statistics [100] to examine the relationship between each M.Yunnan.West population and other populations under the Global Panel B. The program qp3pop implemented in *ADMIXTOOLS* v7.0.2 [101] was applied to calculate the outgroup *f*_*3*_ statistics in the form of *f*_*3*_(Yoruba; X, Y), where X represents the different M.Yunnan.West populations and Y represents other populations under the Global Panel B. The output Z-score was used to measure the genetic affinity between populations.

### Estimation of genetic diversity

To measure the consanguinity of the populations in the NGS Panel, we used *BCFTools* v1.6 to estimate ROH based on the Hidden Markov Model (HMM) approach [102]. We used the *-G* option and set the argument as 30 to account for GT errors. We classified ROH into three classes: short ROH is less than 1 Mb, medium ROH is from 1 to 5 Mb, and long ROH is larger than 5 Mb. Then, we calculated the total length of each type of ROH for each individual and compared ROH length at the population level.

To estimate the IBD-sharing within and between populations, we first used the *Beagle* v5.2 [103] to phase the data of the NGS Panel. Based on the phased data, The IBD segments were estimated using the *hap-IBD* [104], and short gaps in the IBD segments were removed.

Novel variants were defined as those not present in dbsnp v154 [37] and ExAC [38] and were annotated using Ensembl *VEP* v94 [39]. To rule out singletons mainly caused by sample size, we primarily focused on variants excluding singletons. Variants with a SIFT score lower than 0.05 are considered deleterious and variants with a PholyPhen score higher than 0.446 are considered damaging. LOF variants were defined as variants with a high impact classification or missense variants whose SIFT [40] and PolyPhen [41] scores in *VEP* both predicted that the variants were harmful.

### Population history inference

To detect the gene flow of M.Yunnan.West, we used qpDstat in *ADMIXTOOLS* v7.0.2 [101] to calculate *D* statistics by assuming two populations from M.Yunnan.West to be potentially admixed and including the third population from the surrounding populations under Global Panel C as the ancestral population. The Yoruba population from the HGDP dataset was used as the outgroup population. As a result, the tree-like relationship ((X, Y), Z), Yoruba) was used for the detection of gene introgression, where X and Y are target populations to be potentially admixed, and Z is the surrounding population assumed to be the ancestor. Similarly, we also applied qp3pop in *ADMIXTOOLS* to calculate the *f*_*3*_ statistics.

Recent demographic histories were estimated by *IBDNe* v23Apr20 [34]. *IBDNe* was used to estimate estimated the change in effective population size from 1 to 60 generations ago. IBD segments were inferred by *hap-IBD* using the phased data of the NGS Panel. We set IBD segments shorter than the 4 cM that were ignored in *IBDNe*.

Divergence times and migration rates were inferred using the *dadi* v2.1.0 [32] based on the SFS. To avoid the bias caused by the coding sequence regions, we annotated our data using the *VEP* v94 [39] and selected intergenic, synonymous, and intronic sites from the target region as the neutral sites for analysis. As a result, 125,101 SNPs were used to construct pairwise unfolded joint SFS. Ancestral states of variants were annotated using the Enredo-Pecan-Ortheus (EPO) 6-way primate alignment [30]. We used the misidentification function in *dadi* to model the proportion of variants with a misidentified ancestral state. To determine the optimal divergence time and migrations, we ran optimization to infer the best-fit 2-population model parameters from the three given models (Additional file 1: Fig. S16) based on the comparison of the expected and observed SFS in *dadi* (Additional file 1: Fig. S17). We also utilized a 3-step strategy using 3-population models to confirm the topology inferred via pairwise divergence times in 2-population models and the *TreeMix* as the best-fit topology in *dadi* (Additional file 5: Table S4). First, we fixed the TBN as an outgroup population and used different 2-population combinations from the other five populations to test the best-fit topology in each combination. There are 20 different TBN-pop1-pop2 combinations with 3 topologies in each combination (each of the three populations as an outgroup), and we confirmed the topology that TBN is outgroup was the best-fit in all TBN-pop1-pop2 tests. Second, in the remaining five populations, we fixed ACH and JIP and used each of the other three populations (DAI, DEA, and HAN) as the third population to test the best-fit topology in each combination. We found the topology that the third population is the outgroup was the best fit with maximum likelihood in each test. Finally, we used the remaining 3 populations (DAI, DEA, and HAN) to test their topology of them and found that the topology that DEA is the outgroup of DAI and HAN, was the best-fit one. Since *dadi* only supports at most 5 taxa in model construction, after the confirmation of the model topology, we ran the 5-population model using populations except the outgroup TBN to estimate the demographic parameters (Additional file 1: Table S5). For each run of *dadi*, we repeated the optimization process using the parameters from the last round to seed a subsequent round of model fitting, which improves the log-likelihood values and generally converges in the final round. The CIs of demographic parameters in 2-population and 5-population models were estimated by 500 Bootstrapped SFS generated from 100 800-kb blocks containing the target region. Each 800-kb SFS was randomly sampled with replacement and estimated using the same analysis approach described above.

*TreeMix* v1.1.3 [33] was used to confirm the tree topology of the proposed model and infer migration events. We combined the NGS Panel dataset with YRI from KGP [30, 105] and used *PLINK* v1.9 [98] to filter SNPs with a missing rate of less than 0.01. To infer the tree topology, we set the YRI as the outgroup and ran 500 bootstraps in *TreeMix* with no migration. We then applied *SumTrees* from the *DendroPy* [106] o construct a consensus tree based on the 500-bootstrapped trees. To infer migrations, we used the consensus tree as the previously generated tree and ran *TreeMix* with migrations from 1 to 4.

We also used LD-decay to estimate the effective population size and divergence time of populations [35] based on the NGS Panel. We calculated ${r}_{LD}^{2}$ of each pair of SNPs with a genetic distance less than 0.25 cM in each population. Recombination distances were assigned using *PLINK* v1.9 [98] based on the genetic map of HapMap [107]. Effective population sizes of $t$ generations ago were estimated for each population in each recombination distance category as: ${N}_{e}=[(1/{r}_{LD}^{2})-2]*(1/4c)$, where $\mathrm{c}$ denotes the recombination distance. We adjusted ${r}_{LD}^{2}$ as ${r}_{LD}^{2}-(1/n)$, where $n$ is the sample size prior to the calculation of ${N}_{e}$. Divergence times were estimated by ${2N}_{e}{F}_{ST}$, where ${N}_{e}$ is the average of the harmonic means of the relevant recombination distance categories. We used distance categories from 0.01 to 0.25 cM (corresponding to 200 to 5000 generations ago) to estimate ${N}_{e}$ values of target populations.

### Identification of adaptive signals

PBS was applied to detect signals of adaptive evolution in our study. We only included sites with depth above 50 × and a missing rate of less than 5% for PBS calculation. The PBS is defined as:

$${PBS}_{A}=\frac{{T}_{AB}+{T}_{AC}-{T}_{BC}}{2}$$

where $T=-\mathrm{log}(1-{F}_{ST})$, A is the concerned population under selection, and B and C are populations used as references. In calculations, only sites that were polymorphic in at least one of the three populations were considered.

To calculate the significance of PBS values, we performed neutral simulations with *MSMS* v3.2 [108], based on the demographic model inferred in this study. We assumed the divergence time between CEU and East Asians is 2000 generations. Genes with 1–100 SNPs were simulated based on the random sample from all human genes. We then subsampled one million simulations for each number of SNPs per gene (from 1 to 69 SNPs) or using 5-bin categories (from 70 to 100 SNPs). The *P* value of each PBS was defined as the proportion of observed PBS values that were higher than one million simulated PBS values.

To estimate the shared adaptive genes under natural selection, we used HAN in our NGS Panel and CEU from the KGP dataset as the second and third populations for PBS calculation, respectively. We calculated PBS using each M.Yunnan.West population as the target population and focused on the genes with extreme significance (*P* < 0.01) in at least two populations as a shared adaptive signal. Besides, we also used different combinations of M.Yunnan.West population as the target population to examine the relationship between ancestry sharing and PBS distribution.

Divergent adaptation based on PBS was estimated for each M.Yunnan.West population, assuming each of the other three M.Yunnan.West populations as the second population and HAN as the third population. A total of 12 combinations were generated for PBS calculation. Similarly, a gene with extreme significance (*P* < 0.01) in at least two other M.Yunnan.West populations were regarded as a differential adaptive signal.

To validate the differential adaptive genes scanned by the PBS approach, we also estimated cross-population extended haplotype homozygosity (XP-EHH) of extremely significant genes under selection (*p* < 0.01) for each M.Yunnan.West, using *selscan* v1.2.0 [109] and regarding HAN as the reference population.

### Functional analyses of adaptive variants

*VEP* v94 [39] was used to annotate differential adaptive genes with extreme significance (*P* < 0.01). Conservation scores of each variant were calculated by the *–sift* and *–polyphen* options in *VEP*. We also calculated the PBS value of each variant of these genes under selection and listed variants with higher PBS values (> 0.1) as highly differentiated SNPs for each gene.

The gene set was used for functional enrichment of each M.Yunnan.West population was defined as the intersection of strong differential adaptive genes (*P* < 0.05) with the other three M.Yunnan.West populations. Enrichment analysis and PPI network analysis were performed by *metascape* [50] (https://metascape.org), which incorporates popular ontologies for functional enrichment. Functional categories with − log_10_(*P* value) > 2 are displayed as enriched terms across input gene sets. Similar functional categories were classified into one group, and the category with the highest − log(*P* value) is shown in the enrichment plot.

### Supplementary Information

**Additional file 1:** **Fig. S1.** Linguistic distribution of three ancient lineages in Yunnan. **Fig. S2.** Relatedness among samples of each M.Yunnan.West. **Fig. S3.** Principal component analysis (PCA) under the global context. **Fig. S4**. PCA of Global Panel C and M.Yunnan.West. **Fig. S5.** PCA of M.Yunnan.West and their related populations. **Fig. S6**. PCA of DAI from different datasets. **Fig. S7.** Population differentiation measured by *F*_ST_ under the Global Panel C. **Fig. S8.** Outgroup *f3 *statistics of each M.Yunnan.West under the Global Panel B. **Fig. S9.** Unsupervised *ADMIXTURE *analysis from K = 2 to K = 12 under the Global Panel B. **Fig. S10.** Unsupervised *ADMIXTURE *analysis from K = 2 to K = 7 under the Global Panel C. **Fig. S11.** Cross-validation (CV) error of *ADMIXTURE *analysis. **Fig. S12.** Run of homozygosity (ROH) of the populations in the NGS Panel. **Fig. S13.** Identity-by-descendant (IBD) sharing of the populations in the NGS Panel. **Fig. S14.** Correlation of ancestral component with altitude and language family. **Fig. S15.** Potential gene introgression in M.Yunnan.West estimated by *f3 *statistics. **Fig. S16.** 2-population *dadi *models and estimated demographic parameters. **Fig. S17.** Observed and expected site frequency spectrum (SFS) for three 2-population models constructed in *dadi*. **Fig. S18.** Admixture trees estimated using *TreeMix *based on the NGS Panel. **Fig. S19.** Population demography estimated by LD-decay approach under the NGS Panel. **Fig. S20.** Genetic diversity measured by nucleotide differences and novel variants. **Fig. S21.** Novel variants identified in M.Yunnan.West. **Fig. S22.** Adaptive signals with extreme significance validated by cross-population extended haplotype homozygosity (XP-EHH). **Fig. S23.** Gene expression of *SYNC *in GTEx dataset. **Fig. S24.** Functional categories enriched from differential gene set in each of M.Yunnan.West. **Fig. S25.** Protein-protein interaction (PPI) identified from differential gene set in each of M.Yunnan.West. **Table S2.** Information of the populations in Global Panel C. **Table S5.** Demographic parameters of 5-population model with 95% CI estimated by *dadi*. **Table S6.** Number of novel variants of annotation categories defined by *VEP.*

**Additional file 2.** Introduction of studied ethnic minorities.

**Additional file 3:** **Table S1.** Information of samples used in this study.

**Additional file 4:** **Table S3.** Demographic parameters of 2-population models with 95% CI estimated by *dadi*. Pairwise estimations among the populations in Global Panel C were performed using three given models constructed in *dadi.*

**Additional file 5:** **Table S4.** Topology test for demography model using 3-population models in *dadi.*

**Additional file 6:** **Table S7.** List of loss-of-function (LOF) novel variants of M.Yunnan.West populations. LOF novel variants for each M.Yunnan.West were defined as a variant with a high impact classification or a missense variant whose SIFT score is lower than 0.05 and PholyPhen score is higher than 0.446.

**Additional file 7:** **Table S8.** Annotation of major contributing SNPs for adaptive genes with extreme significance. Differential adaptive genes with extreme significance (*P* < 0.01) with major contributing SNPs are shown in the table. Major contributing SNPs were defined as SNPs with PBS values larger than 0.1 in at least one of the other three M.Yunnan.West populations.

**Additional file 8:** **Table S9.** Differential gene set of each M.Yunnan.West population used for enrichment analysis

**Additional file 9:** **Table S10.** Functional categories enriched from differential gene sets using *metascape*. Functional categories with −log_10_(*P*-value) > 2 are displayed as enriched terms across input gene sets. Similar functional categories were classified into one group. Differential gene set for each M.Yunnan.West population in Additional file 8: Table S9 was used as an input gene set for functional enrichment.

## Data Availability

The data underlying this article are available in the National Omics Data Encyclopedia (NODE) at https://www.biosino.org/node and can be accessed with accession number OEP002587. Requests for access to data may be directed to xushua@fudan.edu.cn. All data analyzed during this study are included in this article and its supplementary information files.

## Abbreviations

ACH: Achang
AMM: Asymmetrical migration model
CDS: Coding sequence
CEU: Utah residents with Northern and Western European ancestry
CI: Confidence interval
DAI: Dai
DAF: Derived allele frequency
DEA: Deang
ExAC: Exome Aggregation Consortium
G6PD: Glucose-6-phosphate dehydrogenase
GO: Gene Ontology
GTEx: Genotype-Tissue Expression
HAN: Han Chinese
HGDP: Human Genome Diversity Project
IBD: Identity-by-descendant
ISEA: Island Southeast Asians
JIP: Jingpo
KEGG: Kyoto Encyclopedia of Genes and Genomes
KGP: 1000 Genomes Project
LOF: Loss-of-function
M.Highland: Highland minorities in China
M.North: Minorities in North China
M.South: Minorities in South China
M.Yunnan.West: Minorities in Western Yunnan
MSEA: Mainland Southeast Asians
NGS: Next-generation sequencing
PBS: Population branch statistic
PCA: Principal component analysis
PPI: Protein-protein interaction
ROH: Run of homozygosity
SEA: Southeast Asian
SFS: Site frequency spectrum
SMM: Symmetrical migration model
SNP: Single nucleotide polymorphism
TBN: Tibetan
VEP: Variant Effect Predictor
WES: Whole-exome sequencing
WGS: Whole-genome sequencing
XP-EHH: Cross-population extended haplotype homozygosity
