# Author Correction: Genetic evidence of tri-genealogy hypothesis on the origin of ethnic minorities in Yunnan

**DOI:** 10.1186/s12915-023-01657-4

**Published:** 2023-11-24

**Authors:** Zhaoqing Yang, Hao Chen, Yan Lu, Yang Gao, Hao Sun, Jiucun Wang, Li Jin, Jiayou Chu, Shuhua Xu

**Affiliations:** 1 Department of Medical Genetics, Institute of Medical Biology, Chinese Academy of Medical Sciences, Kunming, 650118 China; 2 Key Laboratory of Computational Biology, Shanghai Institute of Nutrition and Health, University of Chinese Academy of Sciences, Chinese Academy of Sciences, Shanghai, 200031 China; 3 State Key Laboratory of Genetic Engineering, Collaborative Innovation Center for Genetics and Development, Center for Evolutionary Biology, School of Life Sciences, Fudan University, Shanghai, 200438 China; 4 Human Phenome Institute, Zhangjiang Fudan International Innovation Center, and Ministry of Education Key Laboratory of Contemporary Anthropology, Fudan University, Shanghai, 201203 China; 5 Department of Liver Surgery and Transplantation Liver Cancer Institute, Zhongshan Hospital, Fudan University, Shanghai, 200032 China; 6 Center for Excellence in Animal Evolution and Genetics, Chinese Academy of Sciences, Kunming, 650223 China

**Correction: BMC Biol 20, 166 (2022)**

**https://doi.org/10.1186/s12915-022-01367-3**

The original article [1] contains incorrect terms in following sentences.

**Correction 1:**

“Further, the DAI had a high migration rate with HAN, and DEA had a high migration rate with M.Yunnan.West in the same region, including AHC and DAI.”

The abbreviation of Achang in this sentence is incorrect. The “AHC” should be corrected to “ACH”.

**Correction 2:**

“Since our studied populations are different genetic backgrounds and WES data were less used in population genetic studies, we incorporated diverse populations from both whole-genome sequencing (WGS) and genotyping array data like references and designed different dataset panels based on the different analysis purposes.”

In this sentence, “are different genetic backgrounds” should be corrected to “are from different genetic backgrounds”, and “like references” should be corrected to “as references”.

**Correction 3:**

“A few functional mitochondrial alterations and *TAS2R30* might be associated with a higher incidence of hypertension in Di-Qiang populations. Adaptive variants related to malaria and glucose metabolism were identified in the Dai population and indicate the adaptation to thalassemia and G6PD deficiency resulting from malaria resistance, while selection on *PARS2* is likely related to the perception of bitterness.”

The selection signal *TAS2R30* was found in Dai population, and *PARS2* was found in Jingpo population belonging to the Di-Qiang lineage. The “*TAS2R30*” and “*PARS2*” should be interchanged in this sentence.
