# Supplementary material for: Genetic evidence of tri-genealogy hypothesis on the origin of ethnic minorities in Yunnan
Source: BMC Biol. 2022 Jul 21;20:166. doi: 10.1186/s12915-022-01367-3 (PMC9306206; doi:10.1186/s12915-022-01367-3)
Supplement: Supplementary file 1 — Additional file 1: Fig. S1. Linguistic distribution of three ancient lineages in Yunnan. Fig. S2. Relatedness among samples of each M.Yunnan.West. Fig. S3. Principal component analysis (PCA) under the global context. Fig. S4. PCA of Global Panel C and M.Yunnan.West. Fig. S5. PCA of M.Yunnan.West and their related populations. Fig. S6. PCA of DAI from different datasets. Fig. S7. Population differentiation measured by FST under the Global Panel C. Fig. S8. Outgroup f3 statistics of each M.Yunnan.West under the Global Panel B. Fig. S9. Unsupervised ADMIXTURE analysis from K = 2 to K = 12 under the Global Panel B. Fig. S10. Unsupervised ADMIXTURE analysis from K = 2 to K = 7 under the Global Panel C. Fig. S11. Cross-validation (CV) error of ADMIXTURE analysis. Fig. S12. Run of homozygosity (ROH) of the populations in the NGS Panel. Fig. S13. Identity-by-descendant (IBD) sharing of the populations in the NGS Panel. Fig. S14. Correlation of ancestral component with altitude and language family. Fig. S15. Potential gene introgression in M.Yunnan.West estimated by f3 statistics. Fig. S16. 2-population dadi models and estimated demographic parameters. Fig. S17. Observed and expected site frequency spectrum (SFS) for three 2-population models constructed in dadi. Fig. S18. Admixture trees estimated using TreeMix based on the NGS Panel. Fig. S19. Population demography estimated by LD-decay approach under the NGS Panel. Fig. S20. Genetic diversity measured by nucleotide differences and novel variants. Fig. S21. Novel variants identified in M.Yunnan.West. Fig. S22. Adaptive signals with extreme significance validated by cross-population extended haplotype homozygosity (XP-EHH). Fig. S23. Gene expression of SYNC in GTEx dataset. Fig. S24. Functional categories enriched from differential gene set in each of M.Yunnan.West. Fig. S25. Protein-protein interaction (PPI) identified from differential gene set in each of M.Yunnan.West. Table S2. Information of the populations in Global P [file 12915_2022_1367_MOESM1_ESM.pdf]

Genetic evidence of tri-genealogy hypothesis on the origin of ethnic minorities in Yunnan

(Additional file 1)

Zhaoqing Yang,[1] Hao Chen,[2] Yan Lu,[3] Yang Gao,[4] Hao Sun[1], Jiucun Wang,[3,4] Li Jin,[3,4] Jiayou Chu,[1*] Shuhua Xu[3,4,5,6*]

[1]Department of Medical Genetics, Institute of Medical Biology, Chinese Academy of Medical Sciences, Kunming 650118, China

[2]Key Laboratory of Computational Biology, Shanghai Institute of Nutrition and Health, University of Chinese Academy of Sciences, Chinese Academy of Sciences, Shanghai 200031, China

[3]State Key Laboratory of Genetic Engineering, Collaborative Innovation Center for Genetics and Development, Center for Evolutionary Biology, School of Life Sciences, Fudan University, Shanghai 200438, China

[4]Human Phenome Institute, Zhangjiang Fudan International Innovation Center, and Ministry of Education Key Laboratory of Contemporary Anthropology, Fudan University, Shanghai 201203, China

[5]Department of Liver Surgery and Transplantation Liver Cancer Institute, Zhongshan Hospital, Fudan University, Shanghai 200032, China

[6]Center for Excellence in Animal Evolution and Genetics, Chinese Academy of Sciences, Kunming 650223, China

*Corresponding author: Shuhua Xu (Email: xushua@fudan.edu.cn) or Jiayou Chu (Email: chujy@imbcams.com.cn).

This file contains:
Figs. S1-S25, Table S2, Table S5, and Table S6

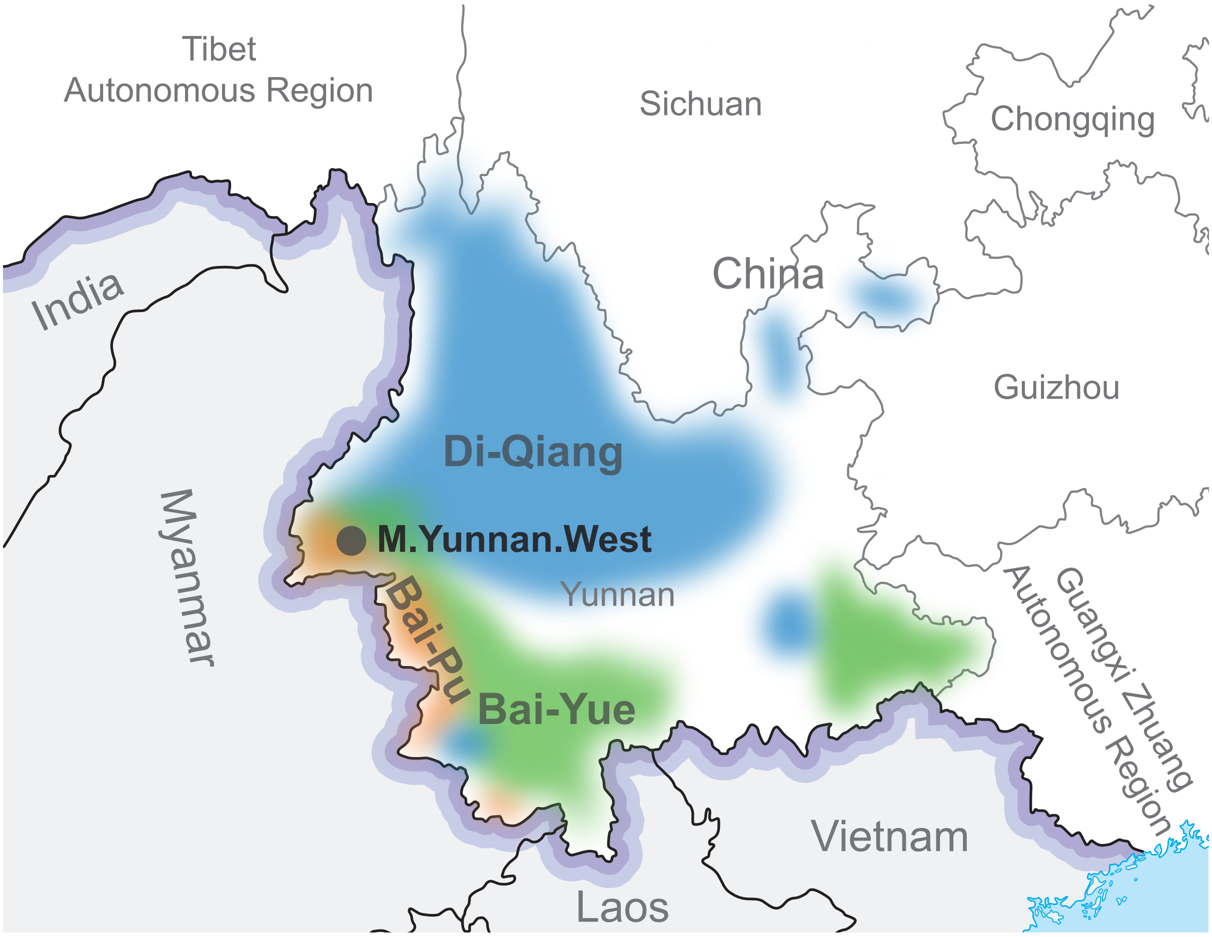

Fig. S1. Linguistic distribution of three ancient lineages in Yunnan.
The linguistically defined distributions of three ancient lineages in Yunnan are illustrated in different colors: blue represents Di-Qiang mainly distributed in Northwestern Yunnan, green represents Bai-Yue mainly distributed in Southwestern Yunnan, and orange represents Bai-Pu distributed in the Western Yunnan that borders with Myanmar. The dot represents the sampling location of western Yunnan minorities. The map used in this figure was obtained from http://bzdt.ch.mnr.gov.cn (GS(2019)1669).

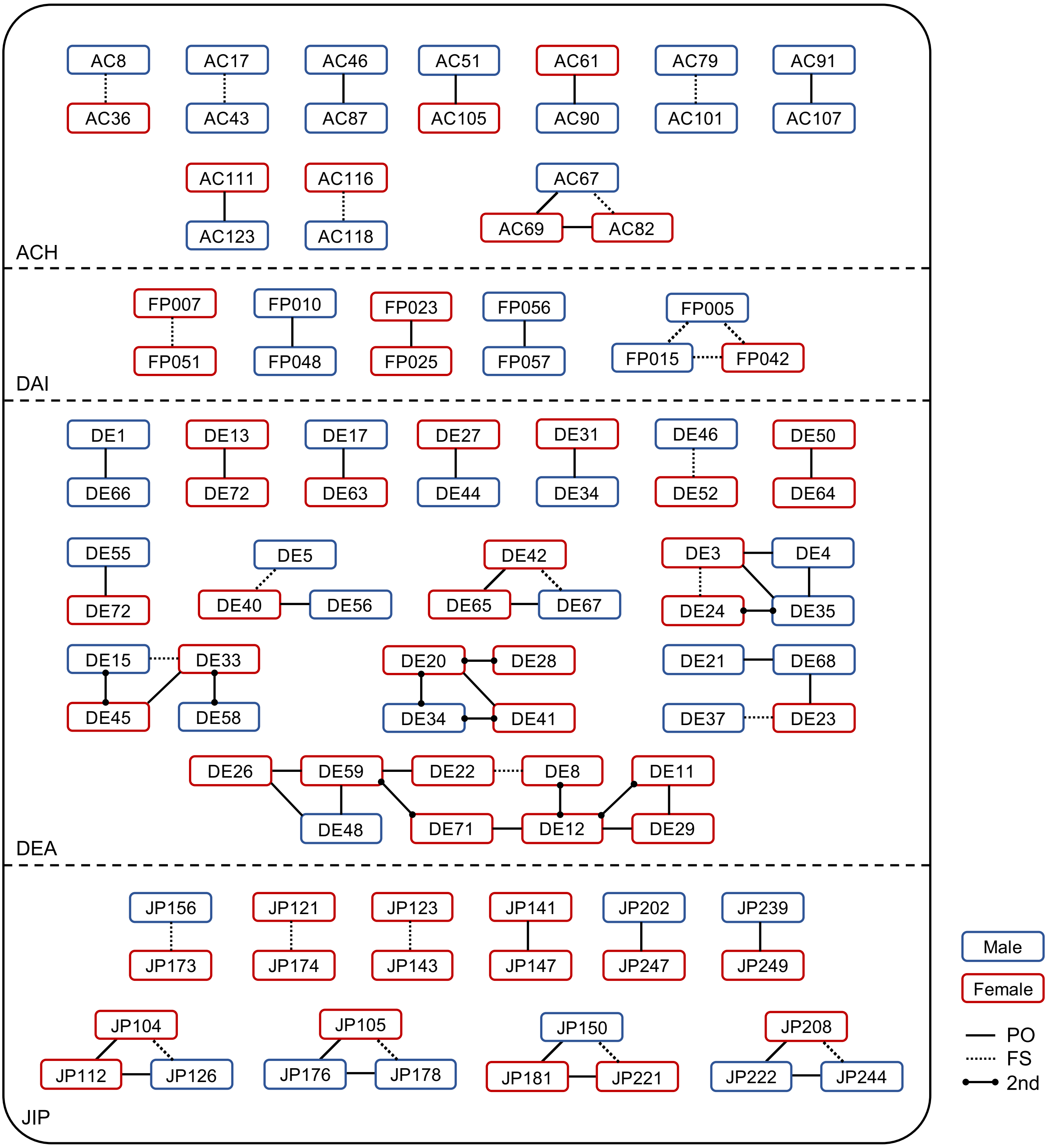

Fig. S2. Relatedness among samples of each M.Yunnan.West.
Graphical representation of the kinship identified by *KING* among male (blue) and female (red) samples of each M.Yunnan.West. Relationship degrees are represented using different types of lines. PO: parent and offspring; FS: full sibling; 2nd: second-degree kinship.

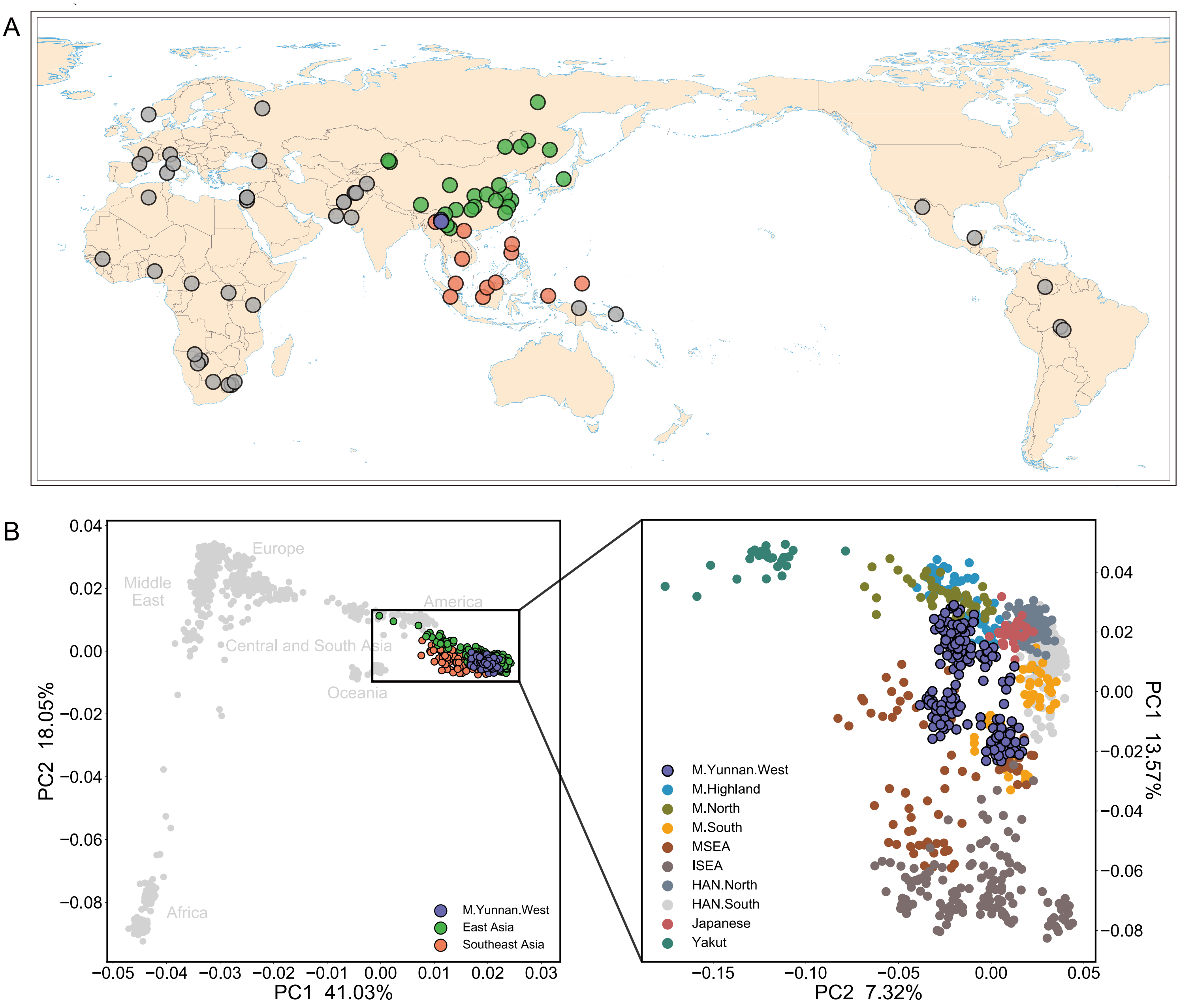

Fig. S3. Principal component analysis (PCA) in the global context.

A: Geographic location of global populations used for PCA, the map used in this figure was obtained from http://bzdt.ch.mnr.gov.cn (GS(2016)1667). B: PCA with 17,101 SNPs of the global populations (1,643 individuals, lower left), and East Asians and Southeast Asians (939 individuals, lower right).

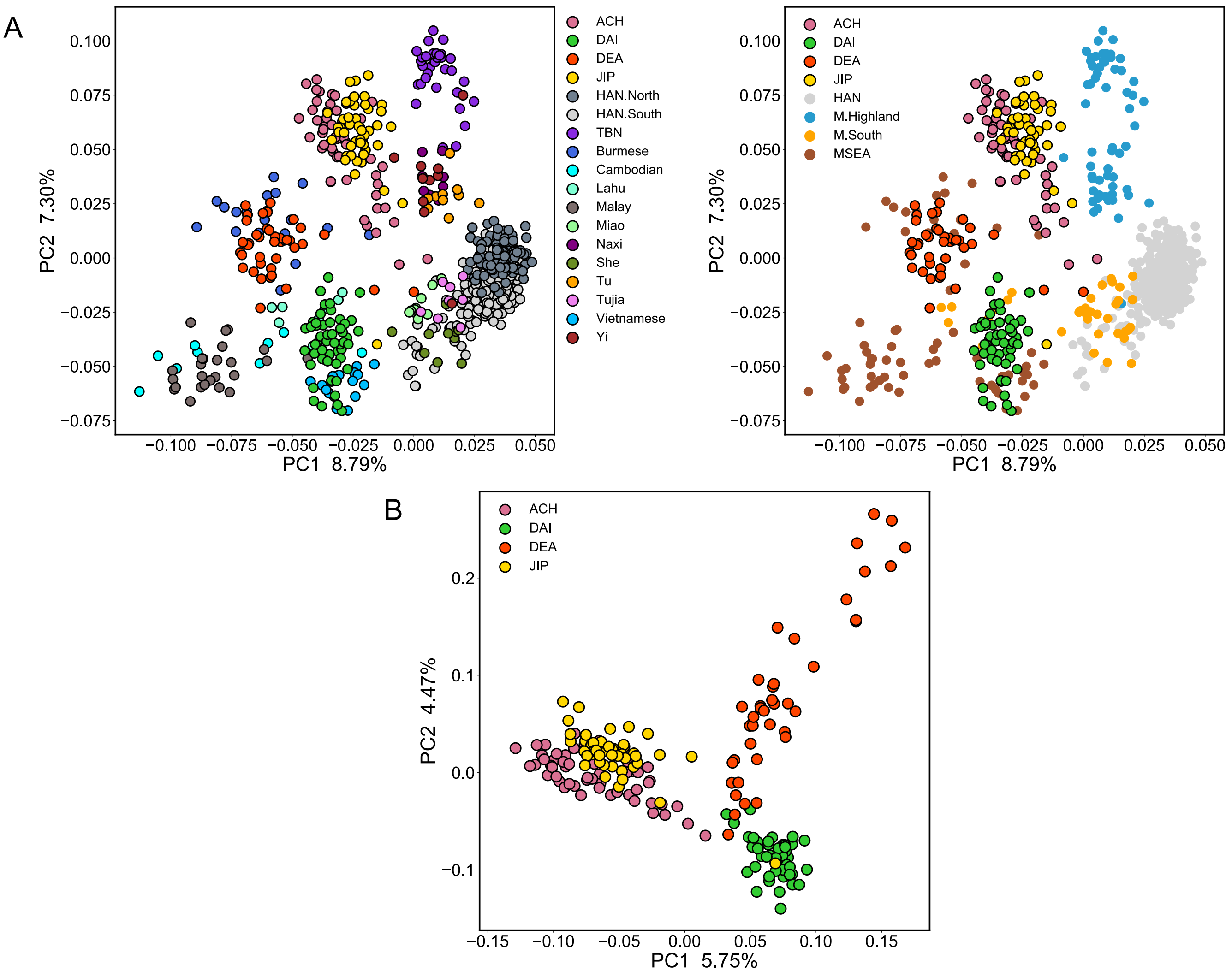

Fig. S4. PCA of Global Panel C and M.Yunnan.West.

PCA performed by a total number of 17,101 SNPs in (A) the populations of Global Panel C (709 individuals) labeled by population names (upper left) and macro-population names (upper right), and (B) M.Yunnan.West (187 individuals).

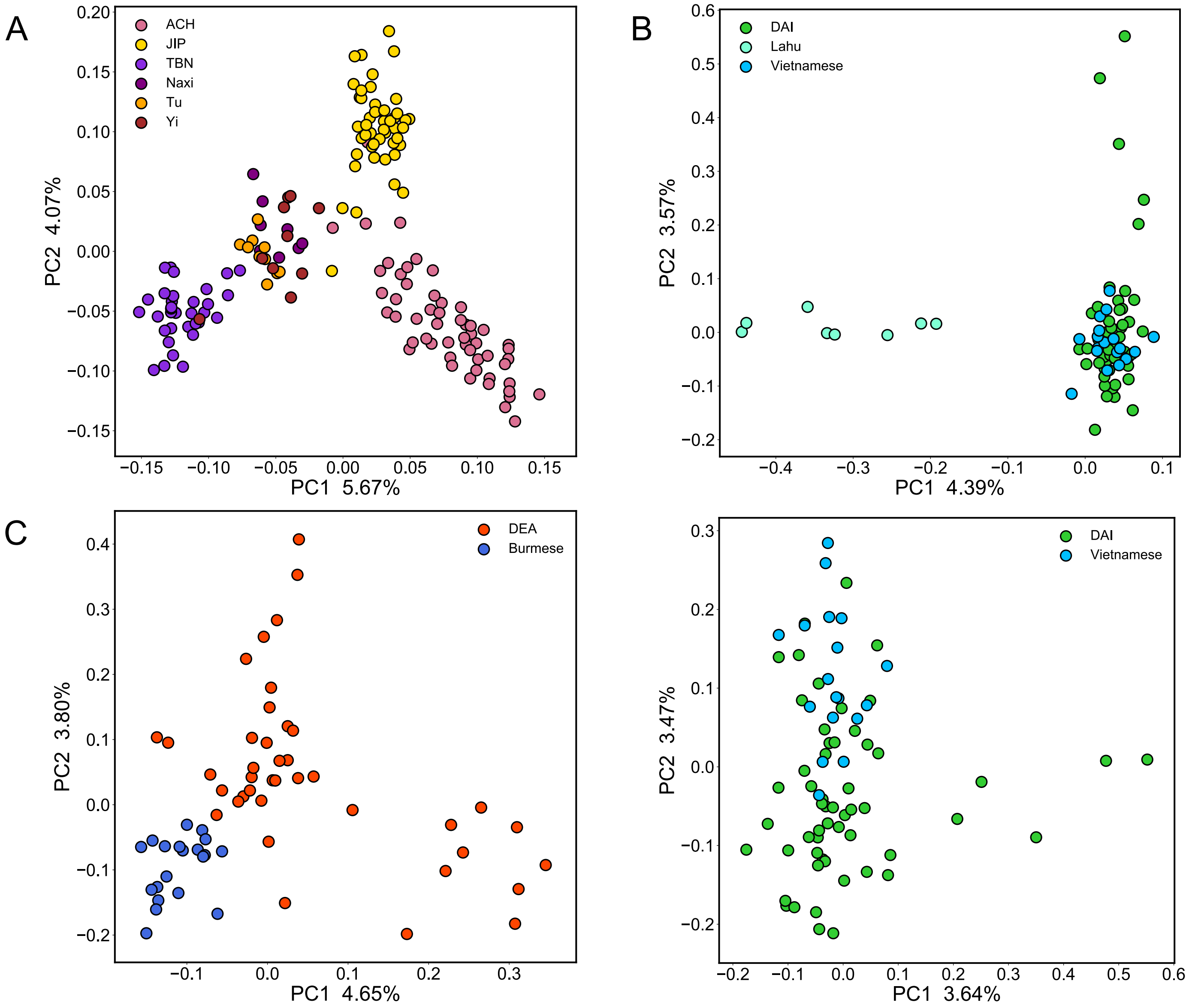

Fig. S5. PCA of M.Yunnan.West and their related populations.
PCA with 17,101 SNPs of (A) ACH and JIP, (B) DAI, (C) DEA, and their respective populations with close affinity. For each subplot, (A) 162, (B) 81 (with Lahu) and 73 (without Lahu), (C) 60 individuals are involved in analysis.

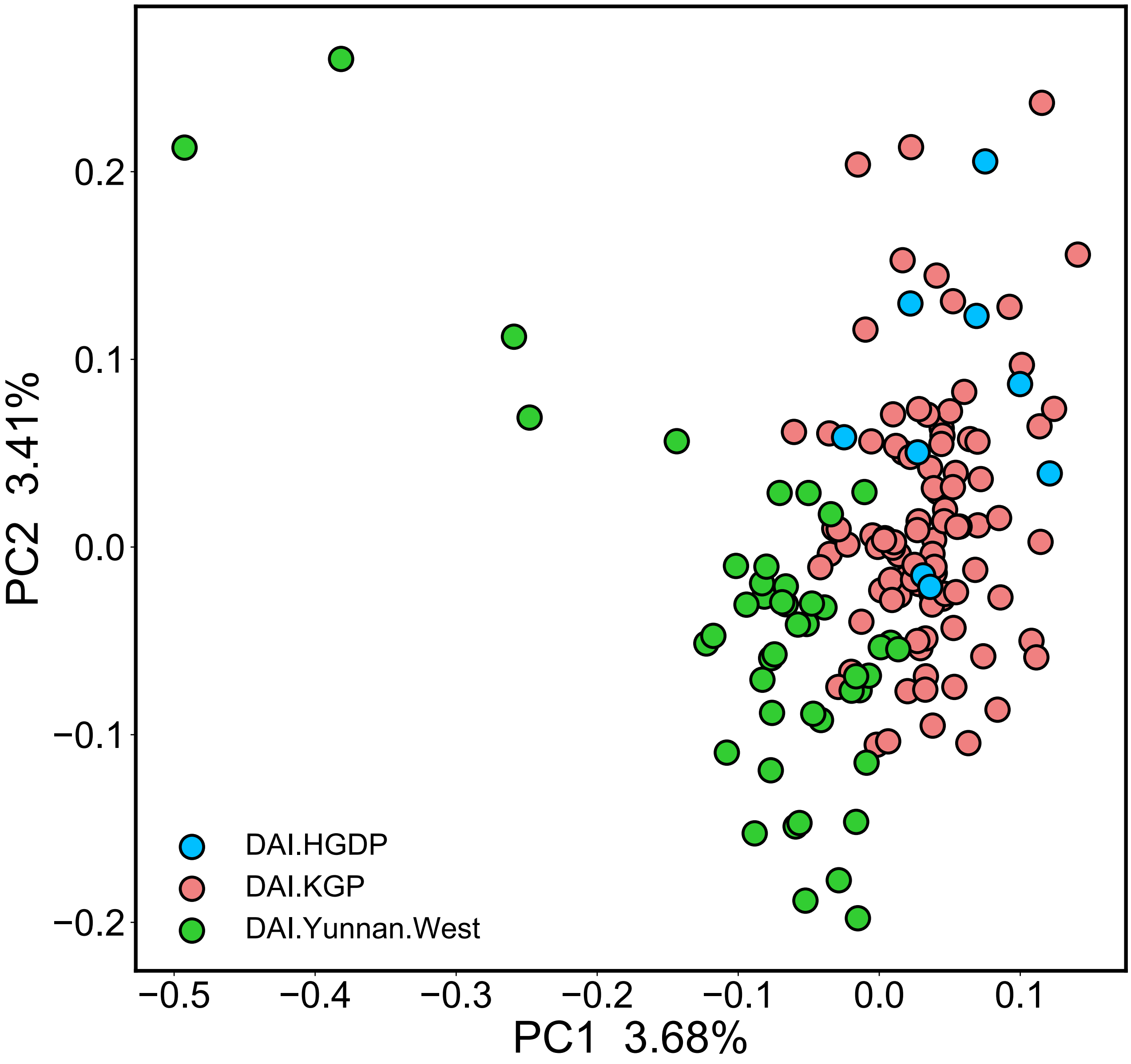

Figure S6. PCA of DAI from different datasets.

PCA performed by a total number of 24,718 SNPs in 148 DAI individuals from this study (green), 1000 Genomes Project (blue) and Human Genomes Diversity Project (pink) dataset.

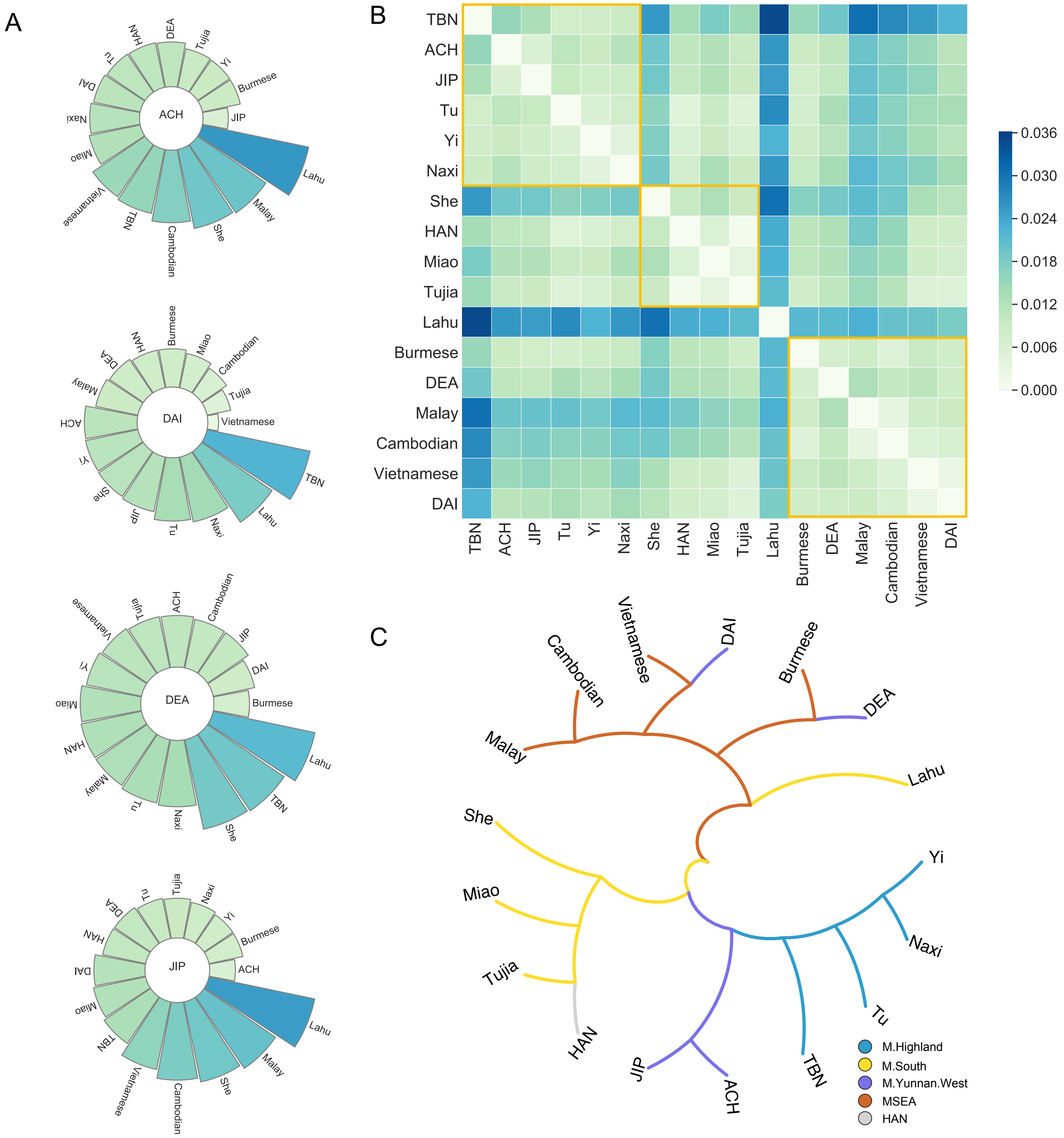

Fig. S7. Population differentiation measured by F_{ST} under the Global Panel C. A: F_{ST} between each M.Yunnan.West and their surrounding populations. B: Heatmap of pairwise F_{ST} among the populations in Global Panel C. C: Population phylogenetic tree based on the result of pairwise F_{ST} in Global Panel C.

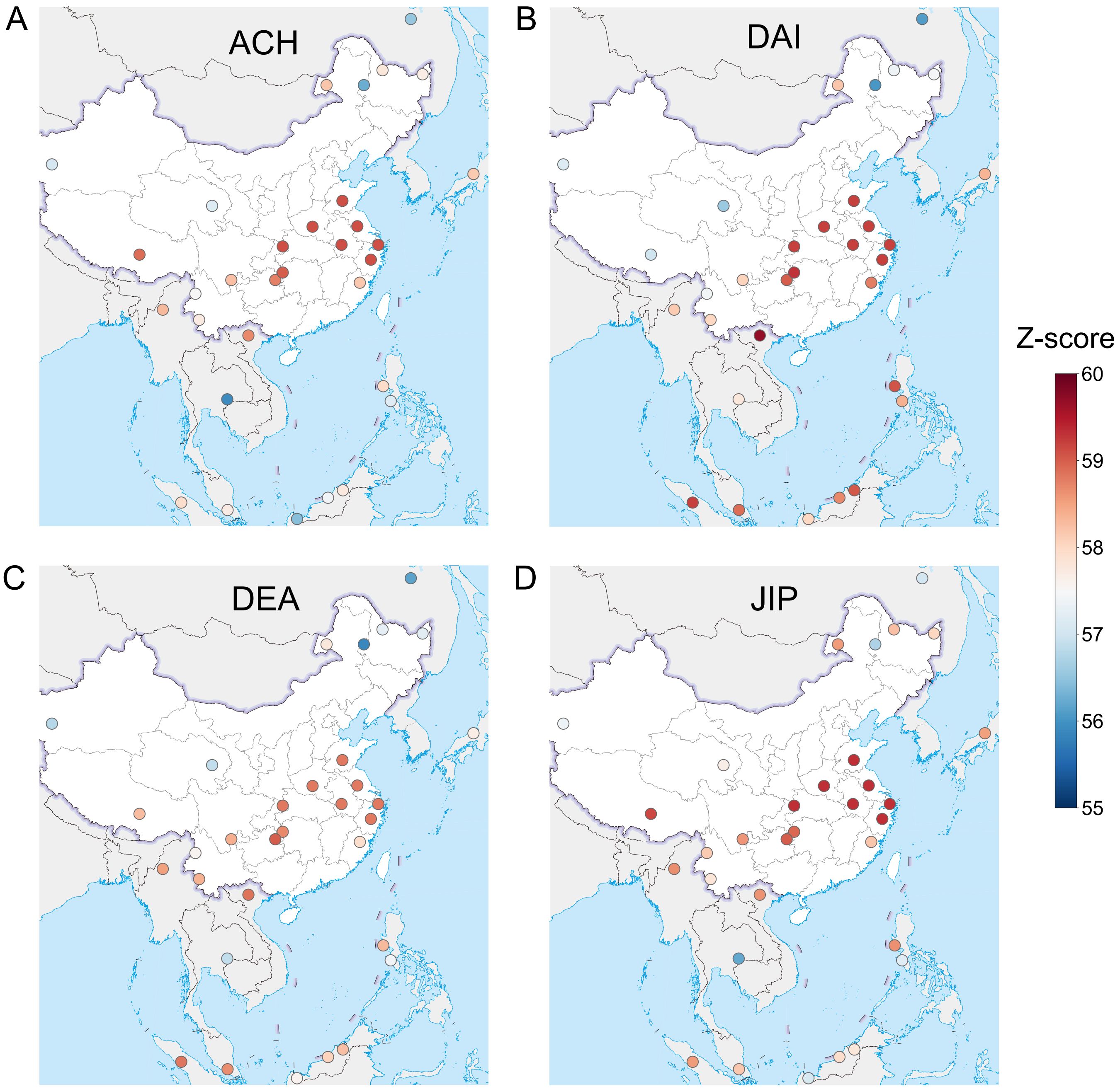

Fig. S8. Outgroup f_3 statistic of each M.Yunnan.West under the Global Panel B. Outgroup f_3 in the form of f_3(Yoruba; X, Y), assuming Y is population from the Global Panel B and X is (A) ACH, (B) DAI, (C) DEA, or (D) JIP. The map used in this figure was obtained from http://bzdt.ch.mnr.gov.cn (GS(2020)4618).

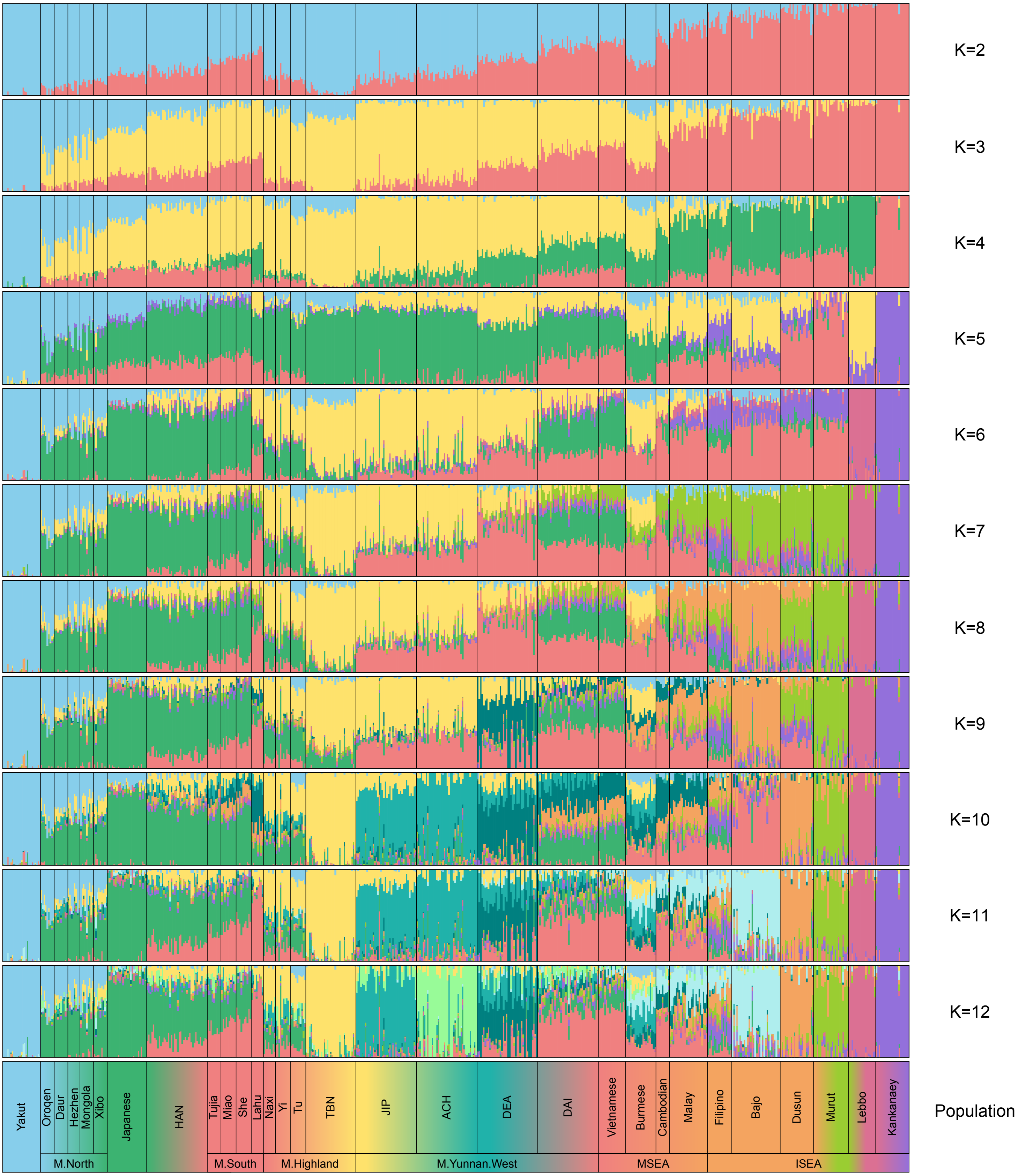

Fig. S9. Unsupervised *ADMIXTURE* analysis from K = 2 to K = 12 under the Global Panel B.

Unsupervised *ADMIXTURE* analysis performed using 28,462 SNPs in 600 samples from the Global Panel B with default parameters. The maximum sample size was restricted to 40 for populations with larger sample sizes.

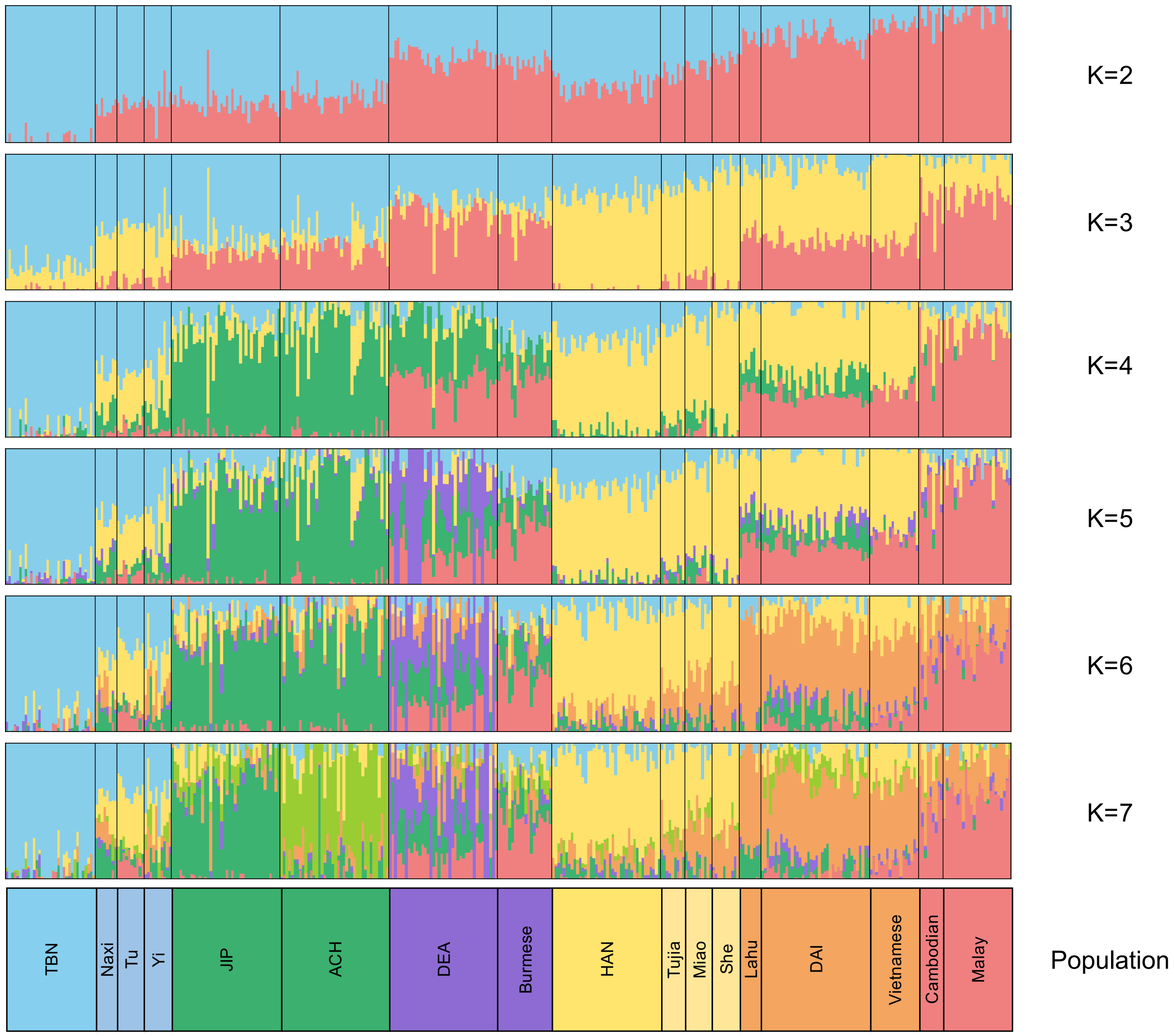

Fig. S10. Unsupervised *ADMIXTURE* analysis from K = 2 to K = 7 under the Global Panel C.

Unsupervised *ADMIXTURE* analysis performed using 28,462 SNPs in 370 samples from the Global Panel C with default parameters. The maximum sample size was restricted to 40 for populations with larger sample sizes.

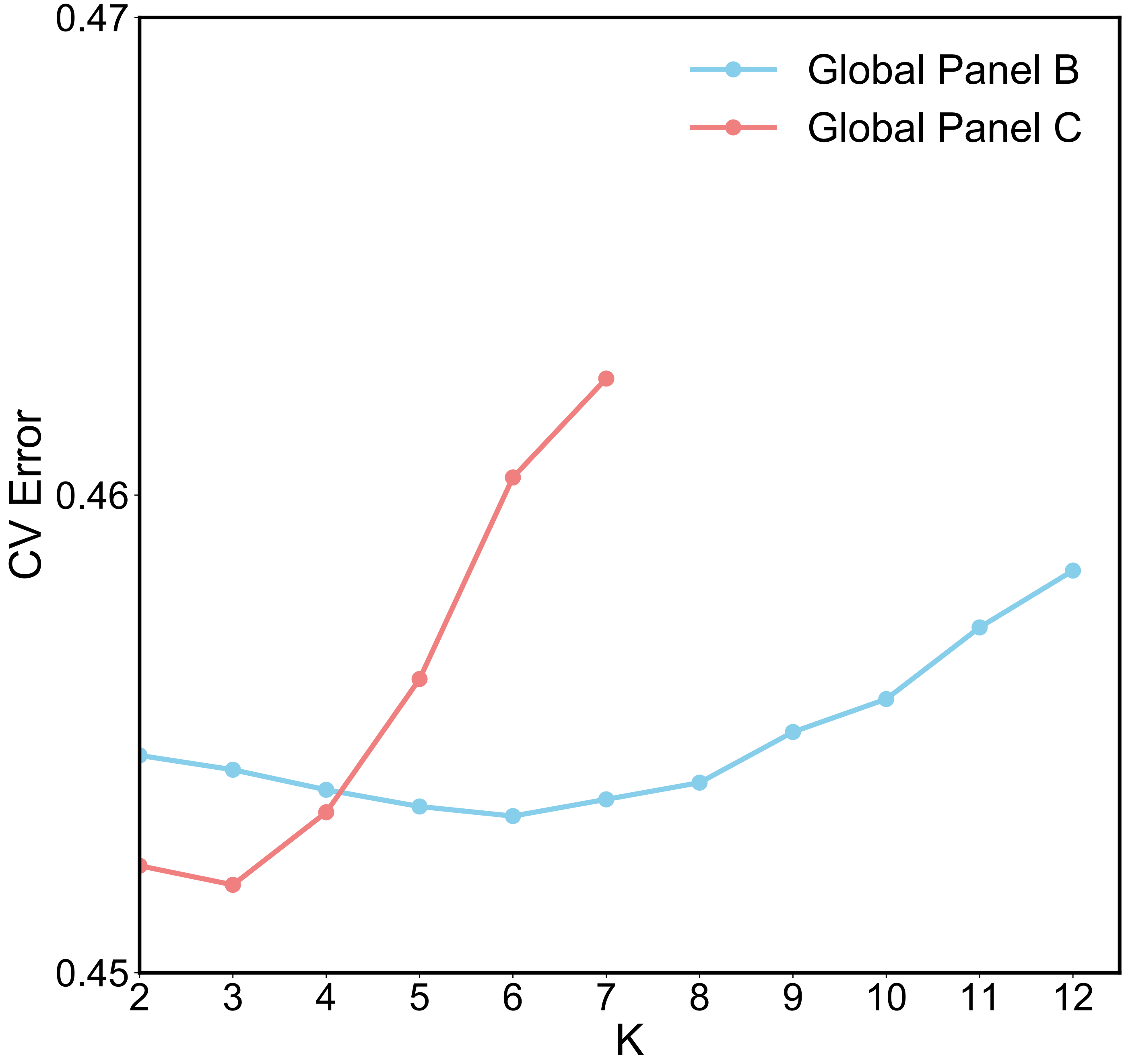

Fig. S11. Cross-validation (CV) error of *ADMIXTURE* analysis.
CV error of *ADMIXTURE* analysis under the Global Panel B (blue) and C (red). The best Ks with lowest CV-error are 6 and 3 for Global Panel B and C, respectively.

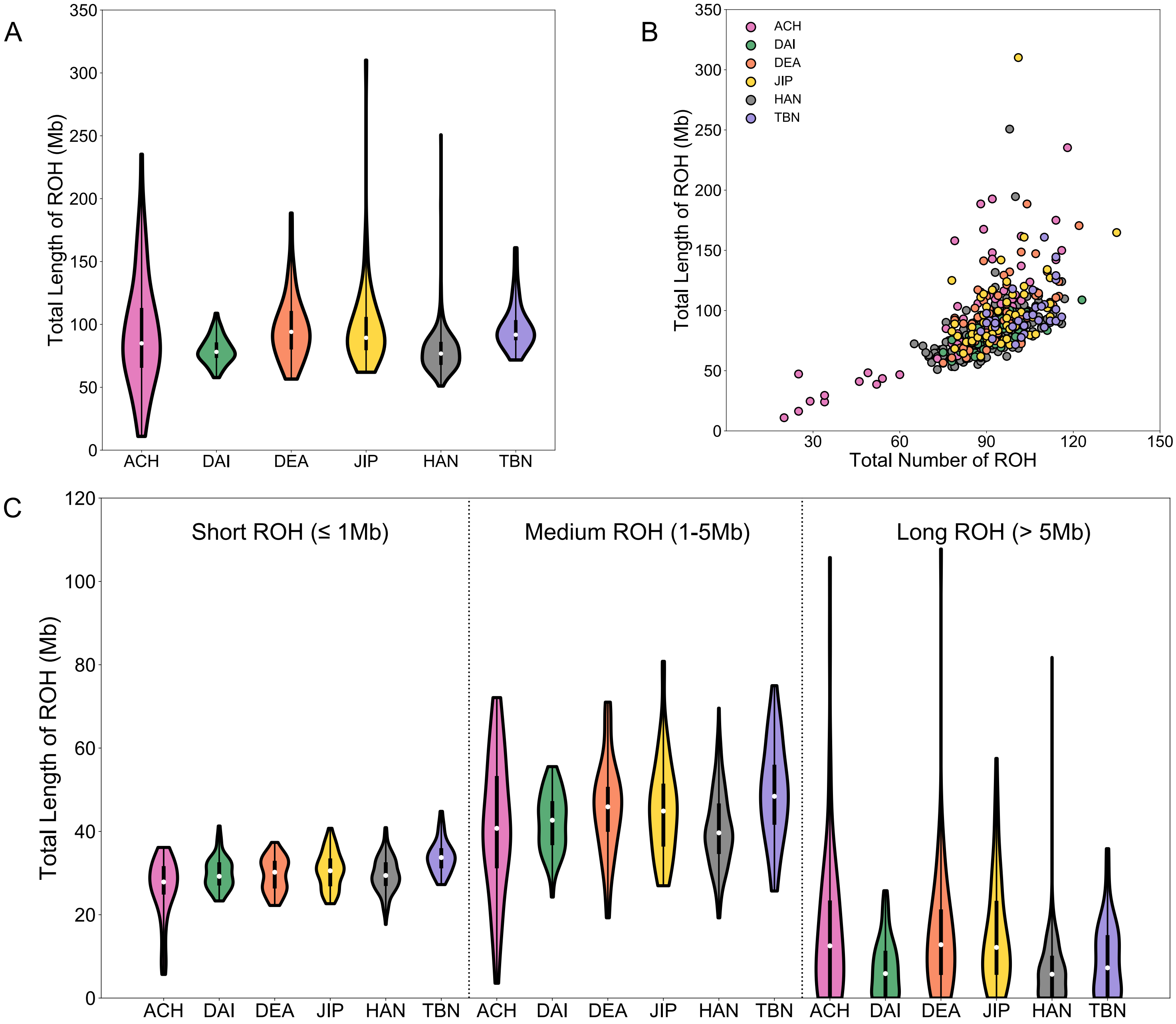

Fig. S12. Run of homozygosity (ROH) of the populations in NGS Panel.
A: The total length of ROH in the populations of NGS Panel. B: Comparison of the total number of ROH (x-axis) and the total length of ROH (y-axis) at individual level. C: The total length of short (≤ 1Mb), medium (1-5 Mb) and long ROH (> 5 Mb) in populations of NGS Panel.

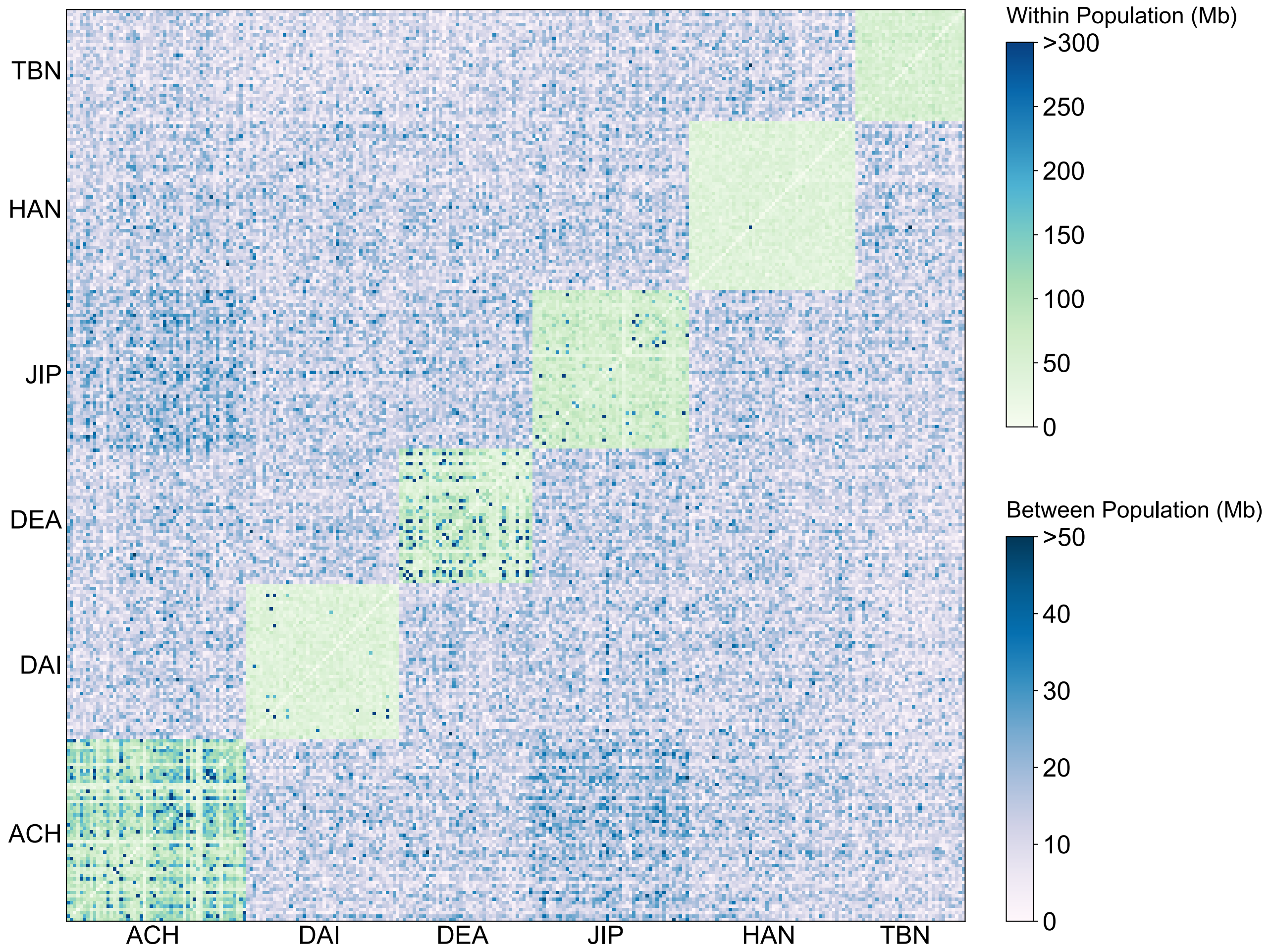

Fig. S13. Identity-by-descendant (IBD) sharing of the populations in NGS Panel. Heatmap of pairwise IBD sharing among the individuals in NGS Panel, estimated by the *hap-IBD*. Two individuals in a pair are the same population or different populations, are defined as within-population and between-population, respectively.

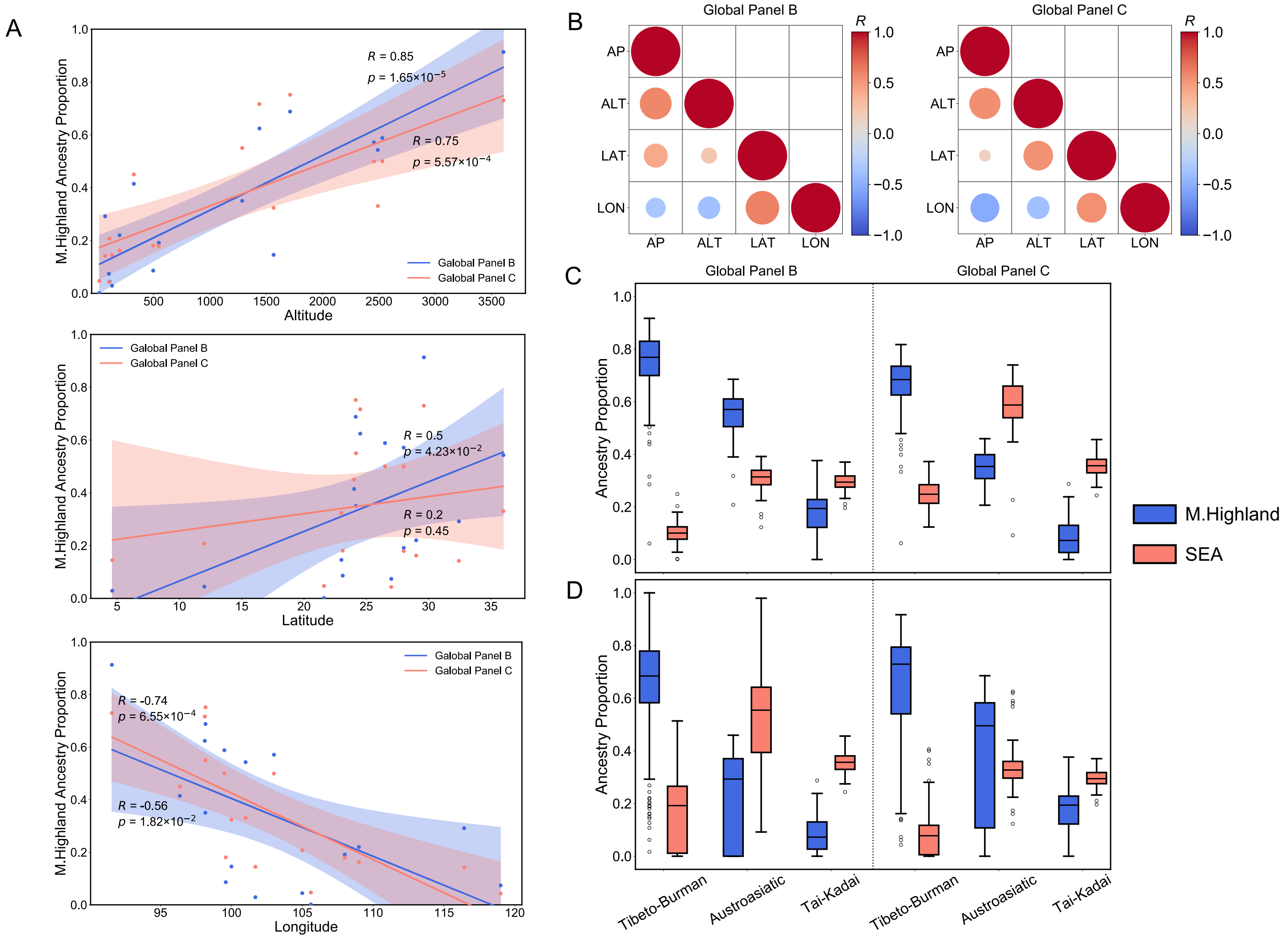

Fig. S14. Correlation of ancestral component with geographical factors and language family.

A: Correlations between proportions of M.Highland ancestry and geographical factors including altitude, latitude, and longitude among the populations in Global Panel C. *ADMIXTURE* results of Global Panel B (Fig. S9, blue) and C (Fig. S10, red) were used for correlation analysis. Region of light color indicate 95% confidence region for each regression fit. B: Partial correlations among M.Highland ancestry proportion and three geographical factors (altitude, latitude, and longitude). The size of circle indicates the $-\log_{10}(P\text{-value})$. AP: M.Highland ancestry proportion; ALT: altitude; LAT: latitude; LON: longitude. C and D: Distribution of proportions of M.Highland (blue) and Southeast Asian (SEA, red) ancestry of three language groups mentioned in tri-genealogy hypothesis. *ADMIXTURE* results of Global Panel B (Fig. S9, left) and C (Fig. S10, right) were both used for the observation of distributions. The B only includes individuals of M.Yunnan.West, while C also includes individuals of surrounding populations in Global Panel C. M.Highland: highlander minorities in China; SEA: Southeast Asians.

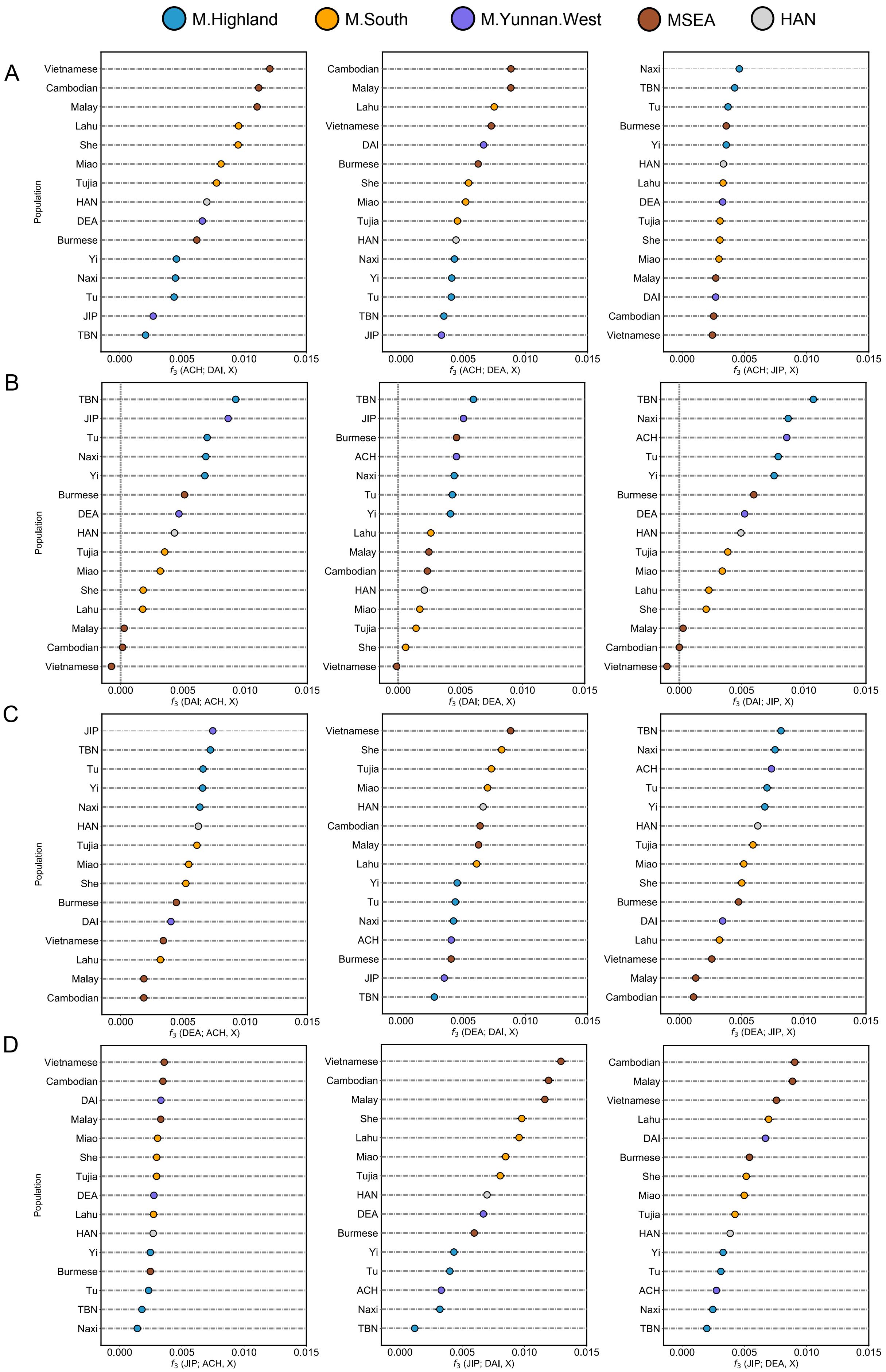

Fig. S15. Potential gene introgression in M.Yunnan.West estimated by *f_3* statistic. *f_3* statistic in the form of *f_3* (X; Y, Z), where X represent the target M.Yunnan.West, including (A) ACH, (B) DAI, (C) DEA, and (D) JIP, Y represent each of the other three M.Yunnan.West, and Z represent populations in Global Panel C.

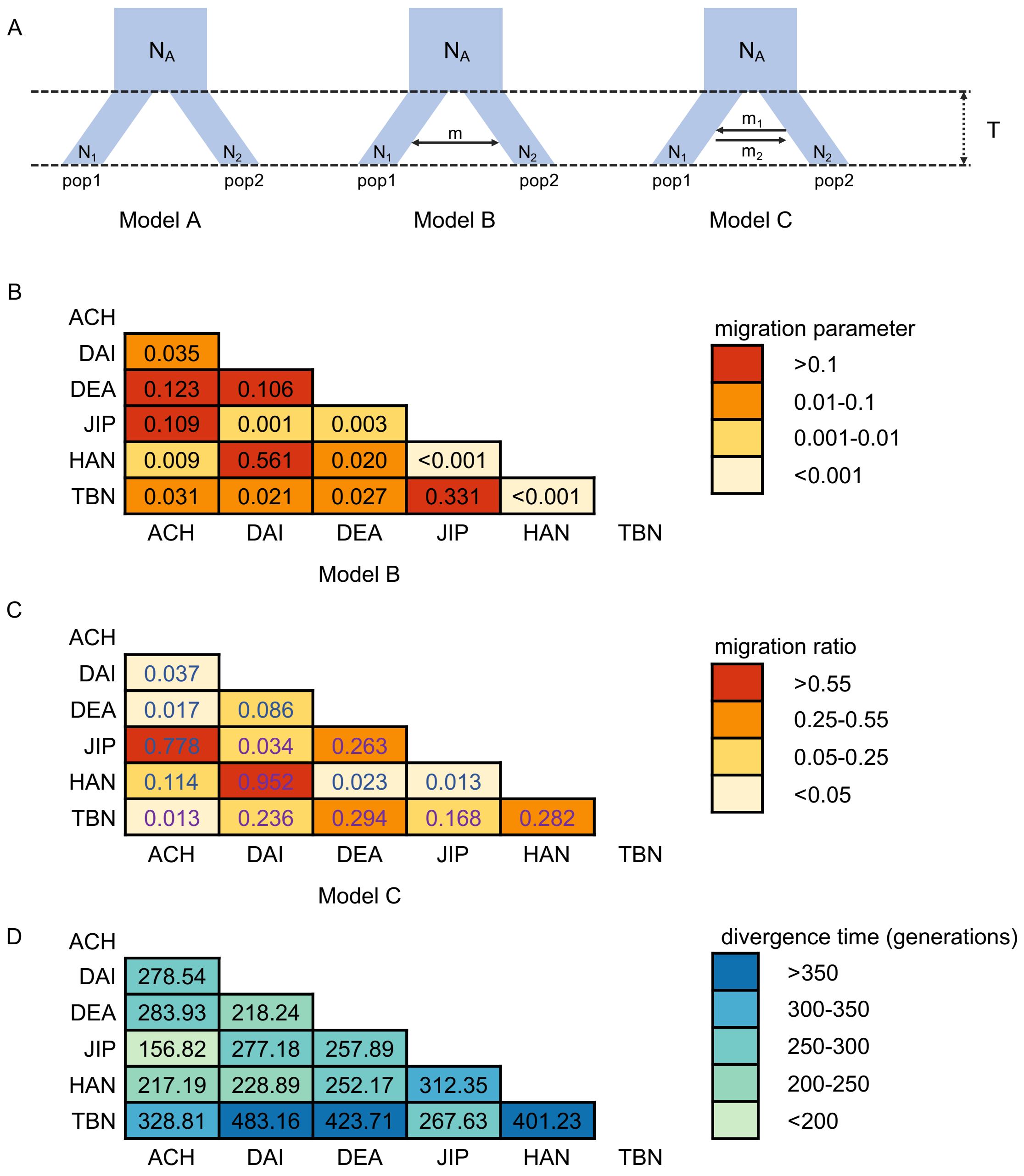

Fig. S16. 2-population dadi models and estimated demographic parameters.

A: Demographic models applied in dadi analysis. B: Migration rate estimated using the model B, the higher migration parameter represents the higher symmetrical migration rate. C: Migration direction estimated using the model C. Each of value is calculated using the higher migration rate over the lower one in pairwise populations, and the higher value indicate the more unbiased gene flow. The blue values indicate the direction from the population in row to the population in column, and the purple values indicate the direction from the population in column to the population in row. D: Pairwise divergence time among the populations in NGS panel, each of divergence time was estimated based on the best-fit parameter from three demographic models.

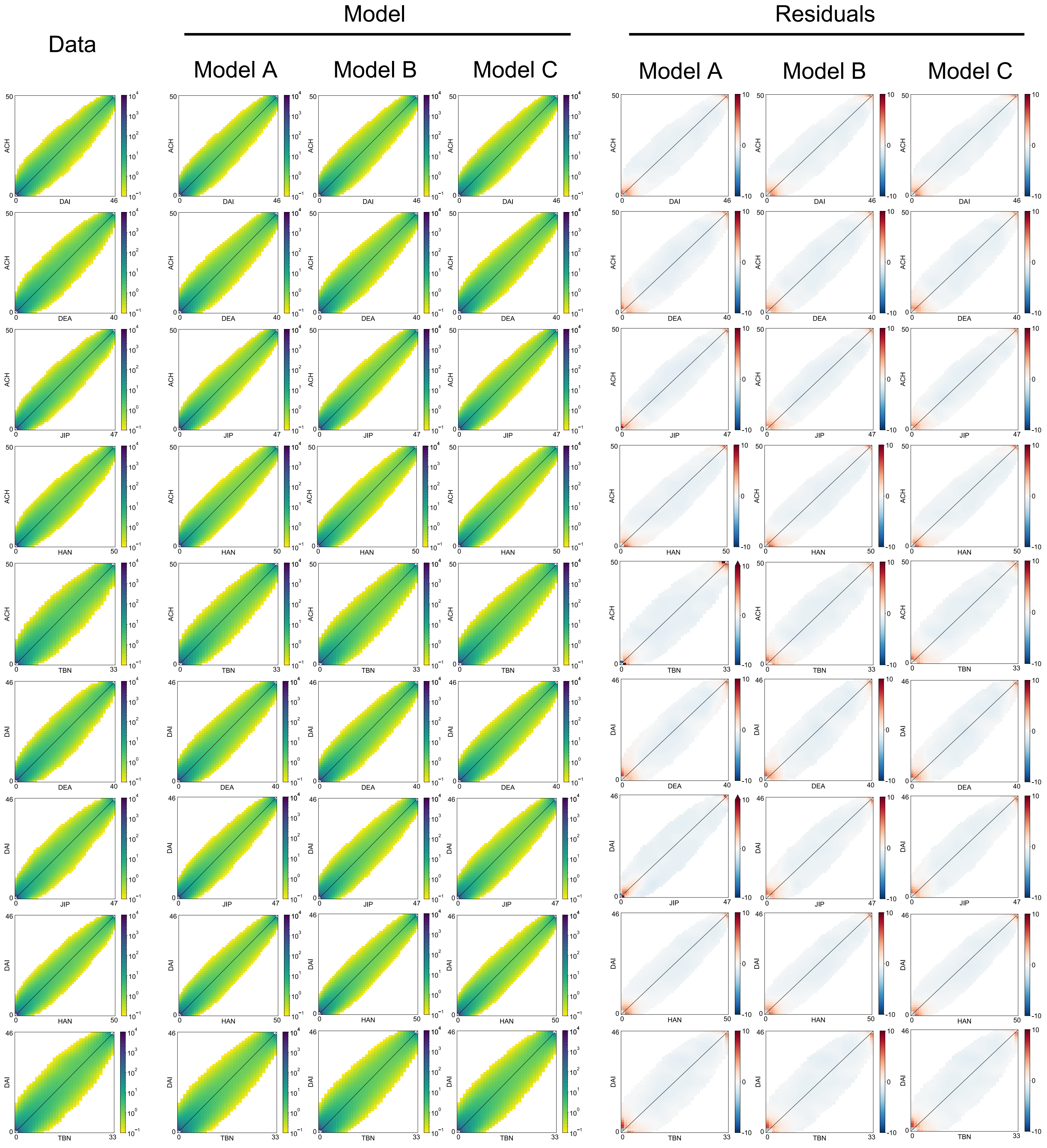
Data
Model
Residuals
Model A
Model B
Model C
Model A
Model B
Model C
ACH
DAI
DEA
JIP
HAN
TBN

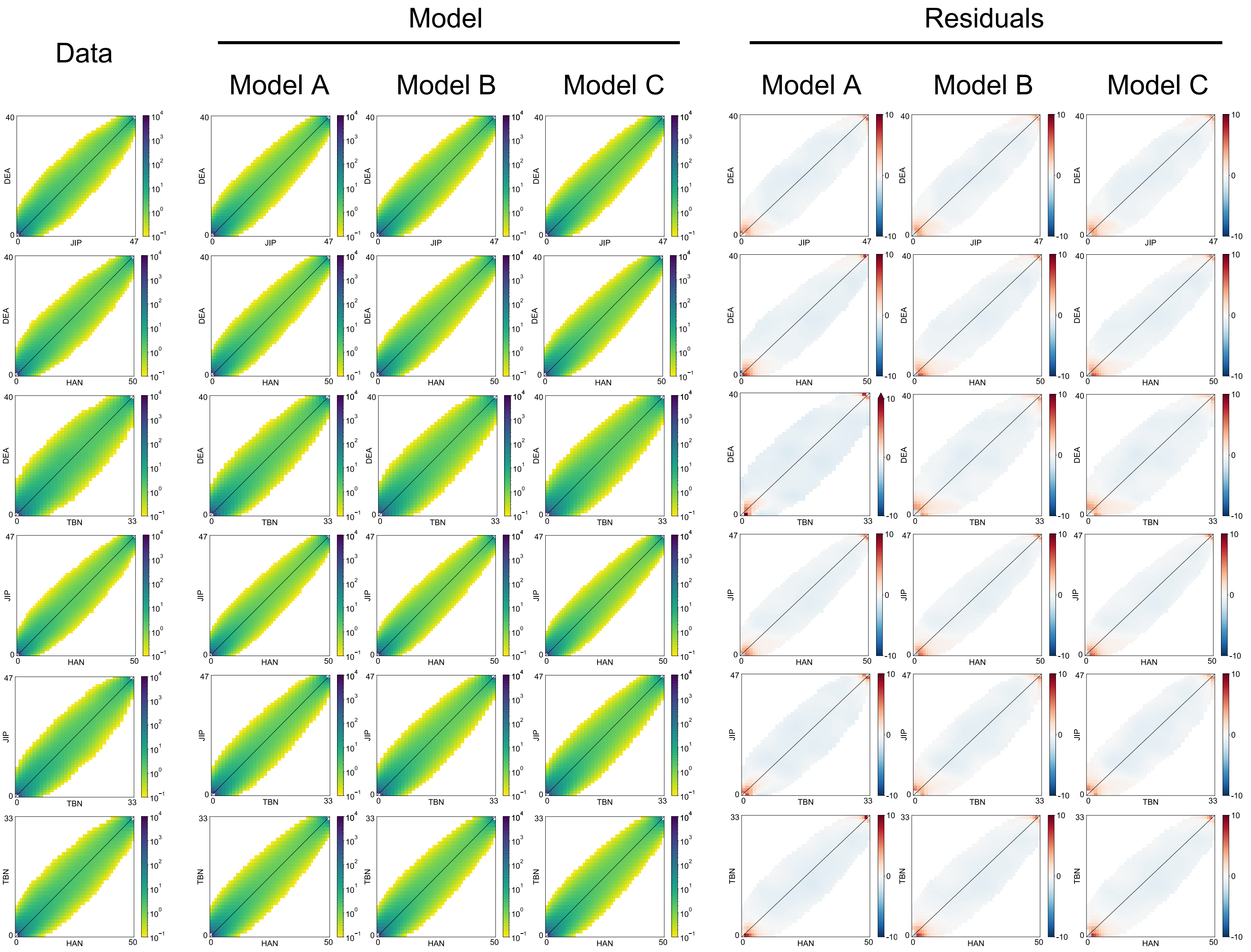

Fig. S17. Observed and expected site frequency spectrum (SFS) for three 2-population models constructed in *dadi*.

Column 1 represents the SFS of observed data, columns from 2 to 4 represent the model-based predicted SFS, columns from 5 to 7 represent the residuals of model minus data. Each row represents the pairwise comparison among the populations in NGS Panel.

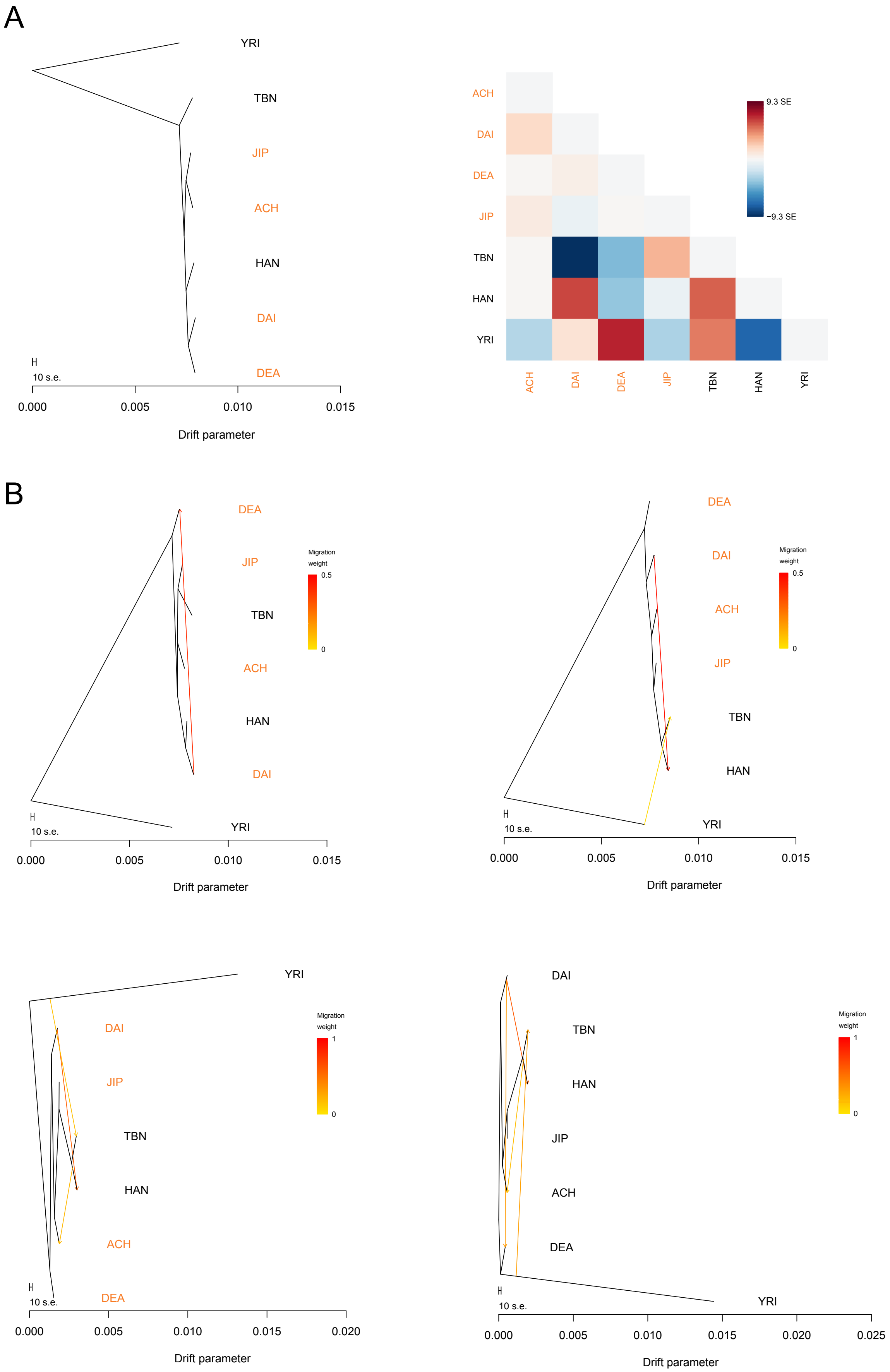

Fig. S18. Admixture trees estimated using *TreeMix* based on the NGS Panel.
A: Tree topology without migration events inferred by maximum likelihood, along with residual matrices. B: Admixture trees with migration events from 1 to 4.

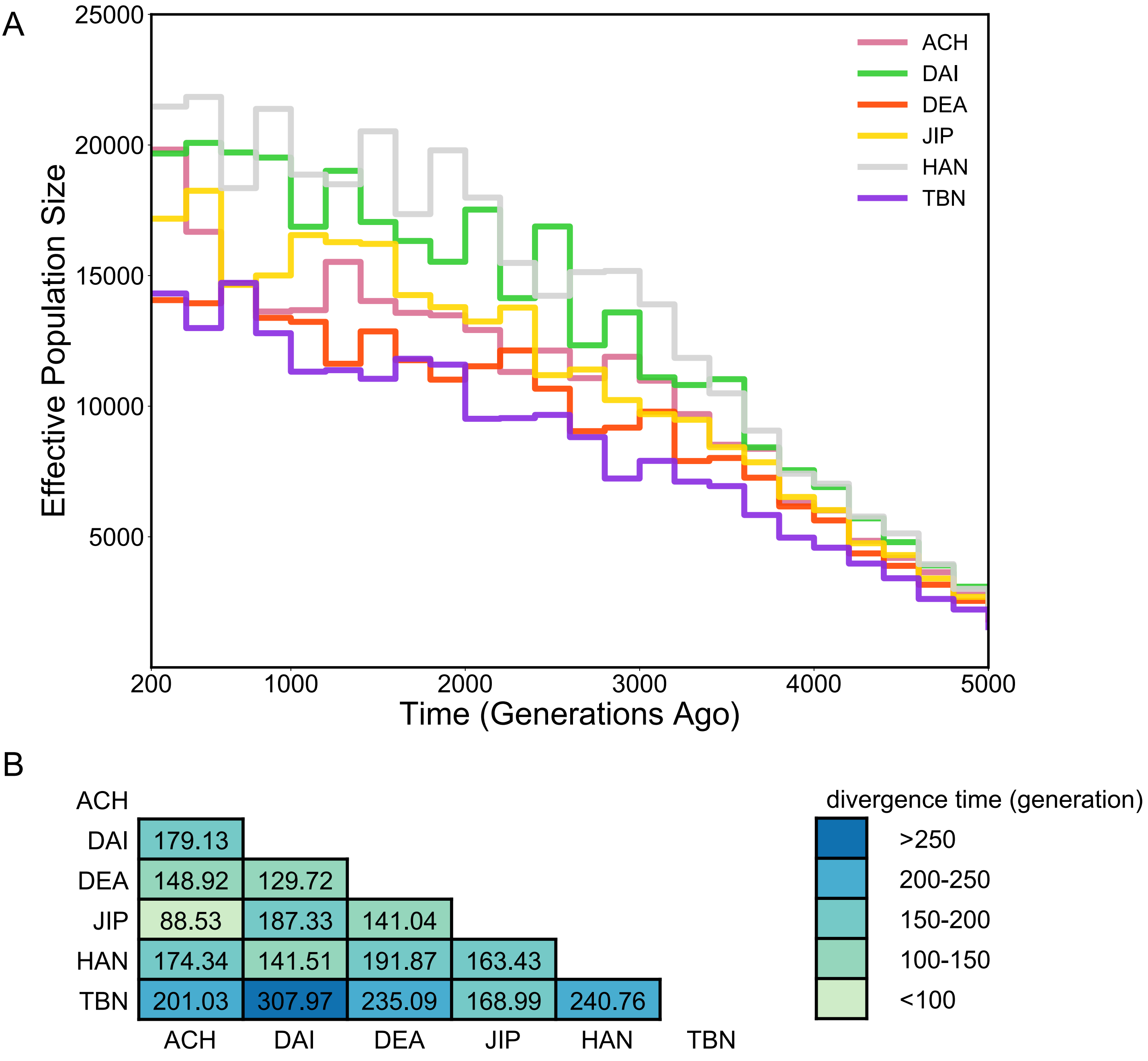

Fig. S19. Population demography estimated by LD-decay approach under the NGS Panel.
A: Effective population size (N_e) estimated for each population based on LD observations in 250 classes from 0.01 cM to 0.25 cM (corresponding to 200 to 5,000 generations ago) with the window size set as 0.001 cM. B: Pairwise divergence time measured by the harmonic mean of effective population sizes in (A) and pairwise F_{ST} estimated in Fig. S7.

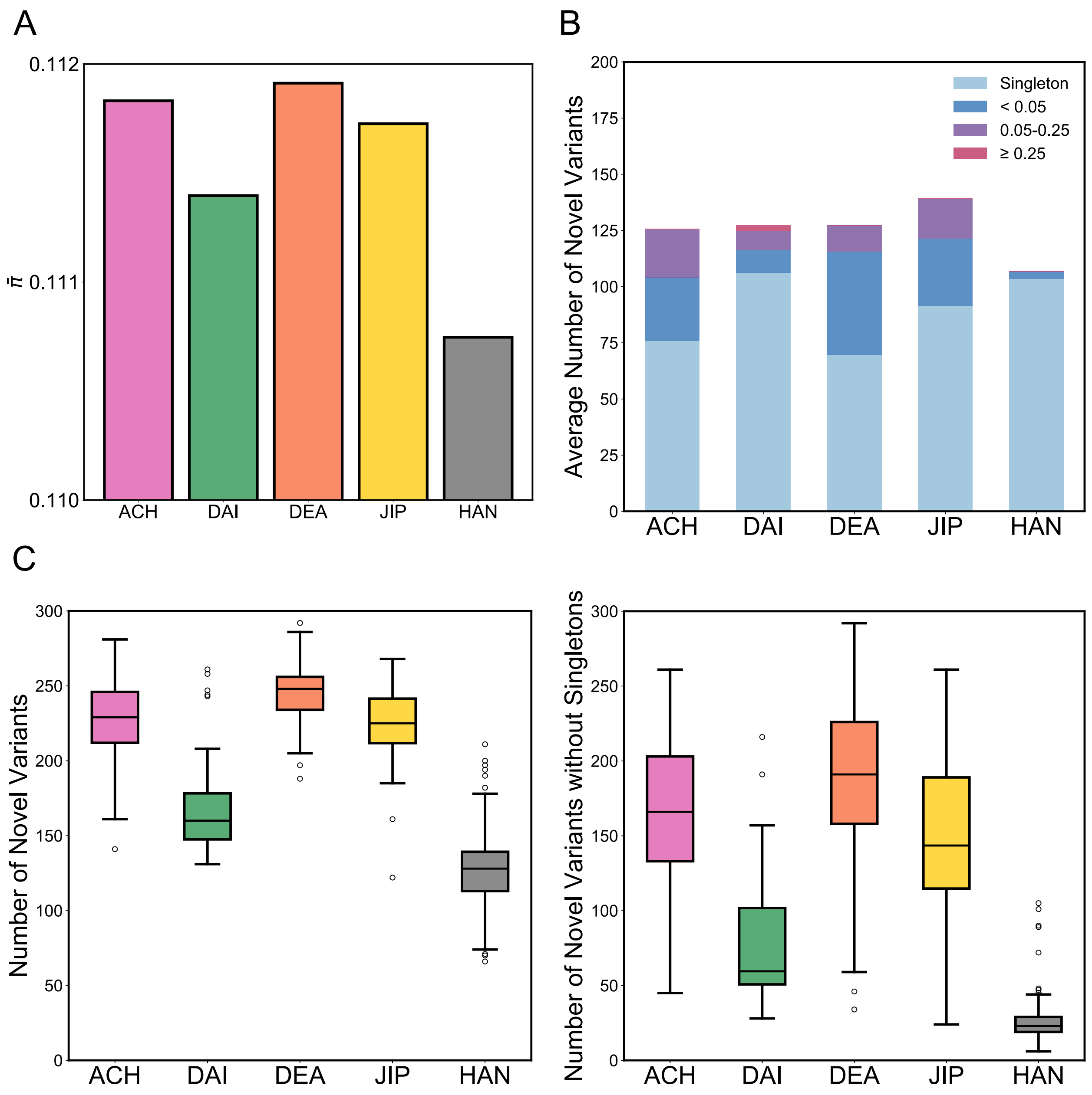

Fig. S20. Genetic diversity measured by nucleotide differences and novel variants.

A: Average nucleotide differences ($\bar{\pi}$) in exome target region of each M.Yunnan.West. B: Number of novel variants with allele frequency classification. C: Boxplot representing the distribution of the number of novel variants at individual level for each M.Yunnan.West, including (left) or excluding (right) singletons.

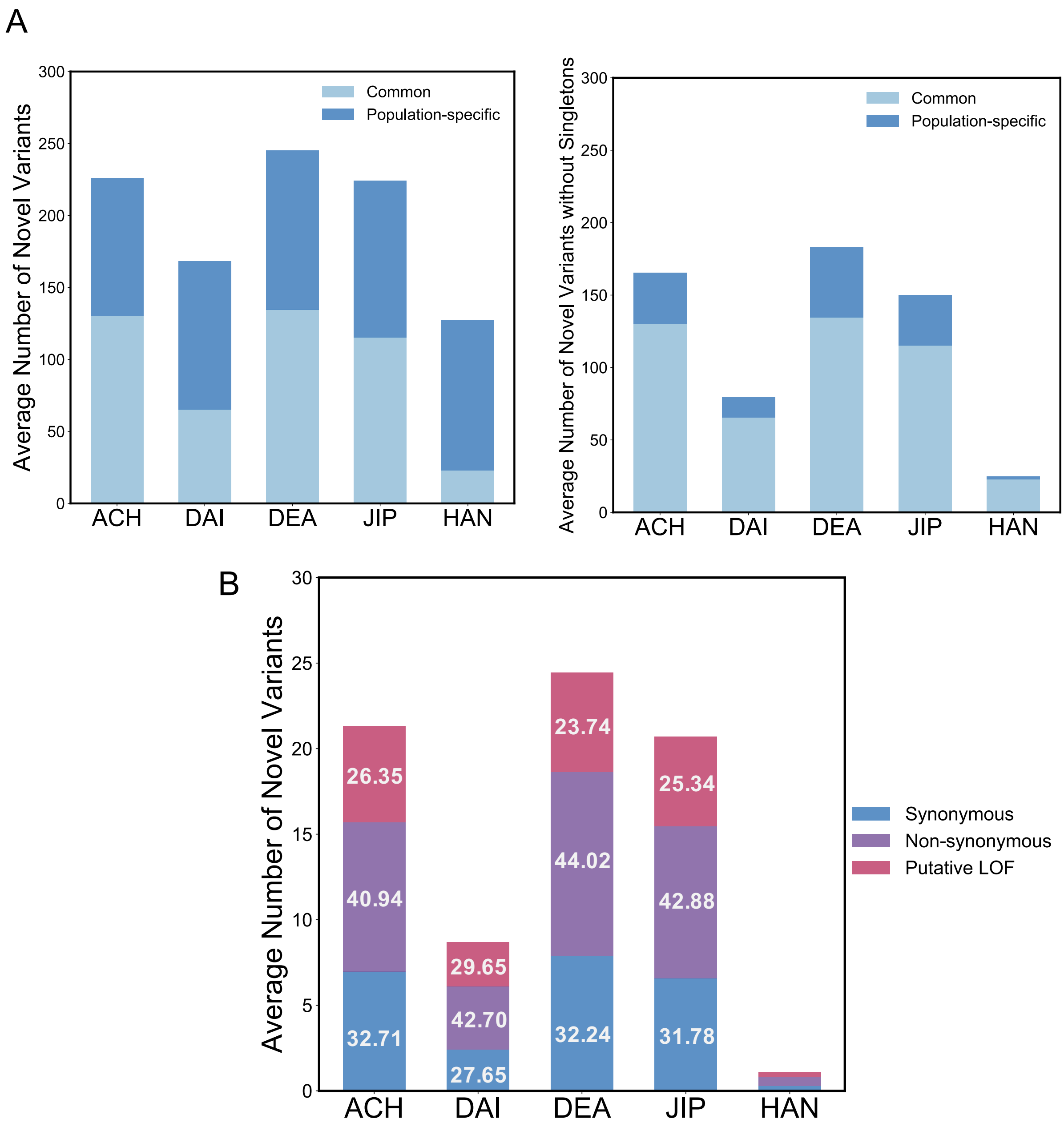

Fig. S21. Population-specific and functional novel variants identified in M.Yunnan.West.

A: The number of common and population-specific novel variants discovered in each population, including (left) or excluding (right) singletons. B: Variant consequences of novel variants in CDS region annotated by Ensembl Variant Effect Predictor (*VEP*). Loss-of-function (LOF) variant was defined as a variant with a high impact classification or missense variant whose SIFT and PolyPhen scores are both predicted-damaging in *VEP*. White values represent the proportion of annotation categories.

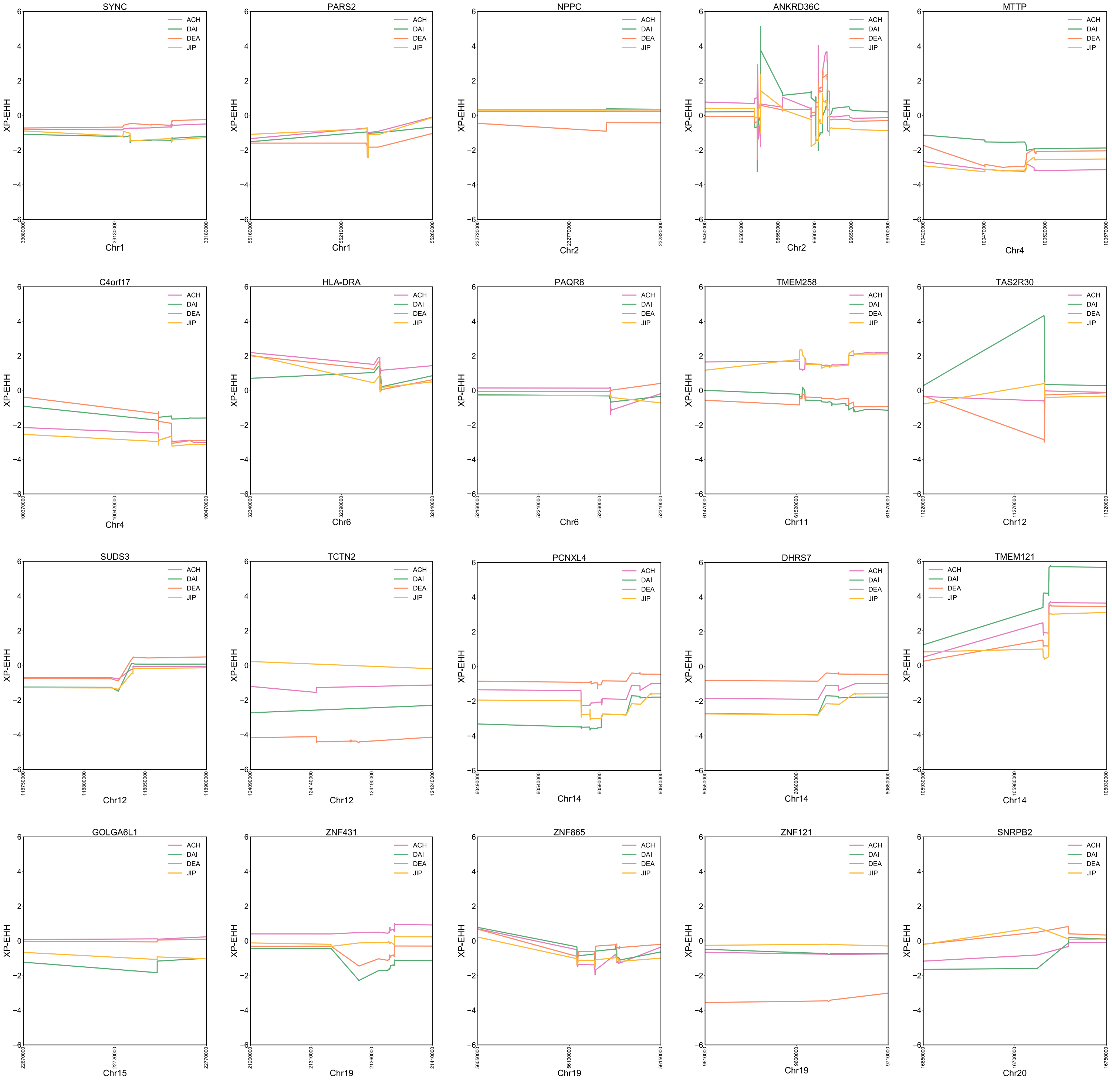

Fig. S22. Adaptive signals with extreme significance validated by the cross-population extended haplotype homozygosity (XP-EHH).

Differential adaptive signals for each M.Yunnan.West detected by PBS were also scanned using XP-EHH. Analysis of XP-EHH was performed by *selscan*, using HAN as reference population. 16 of 27 genes show evident selection signatures in XP-EHH.

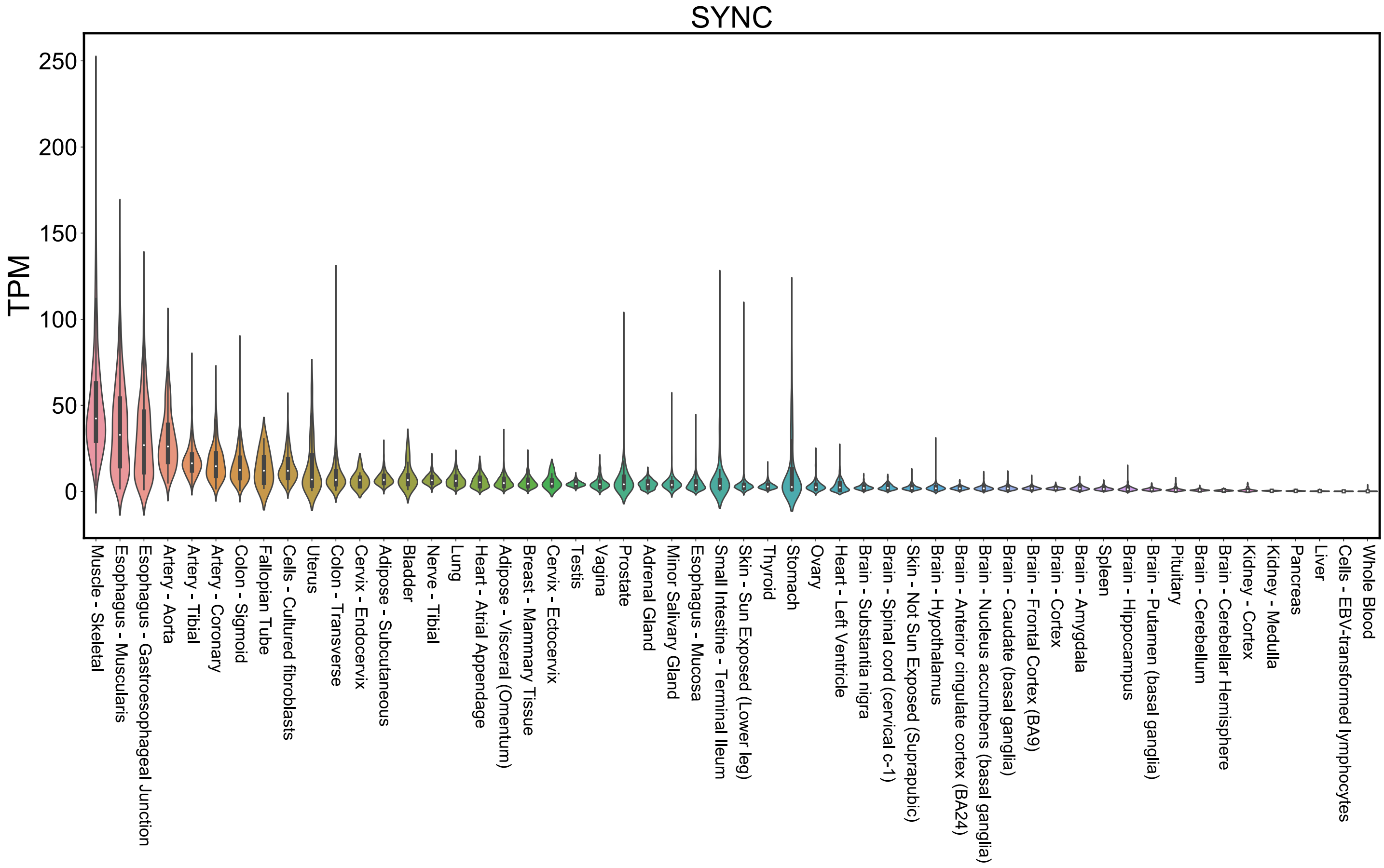

Figure S23. Gene expression of *SYNC* in the GTEx dataset.

Multi-tissue gene expression of *SNYC* based on the GTEx dataset. The x-axis represents different tissues and y-axis represents gene expression level in Transcripts Per Million (TPM).

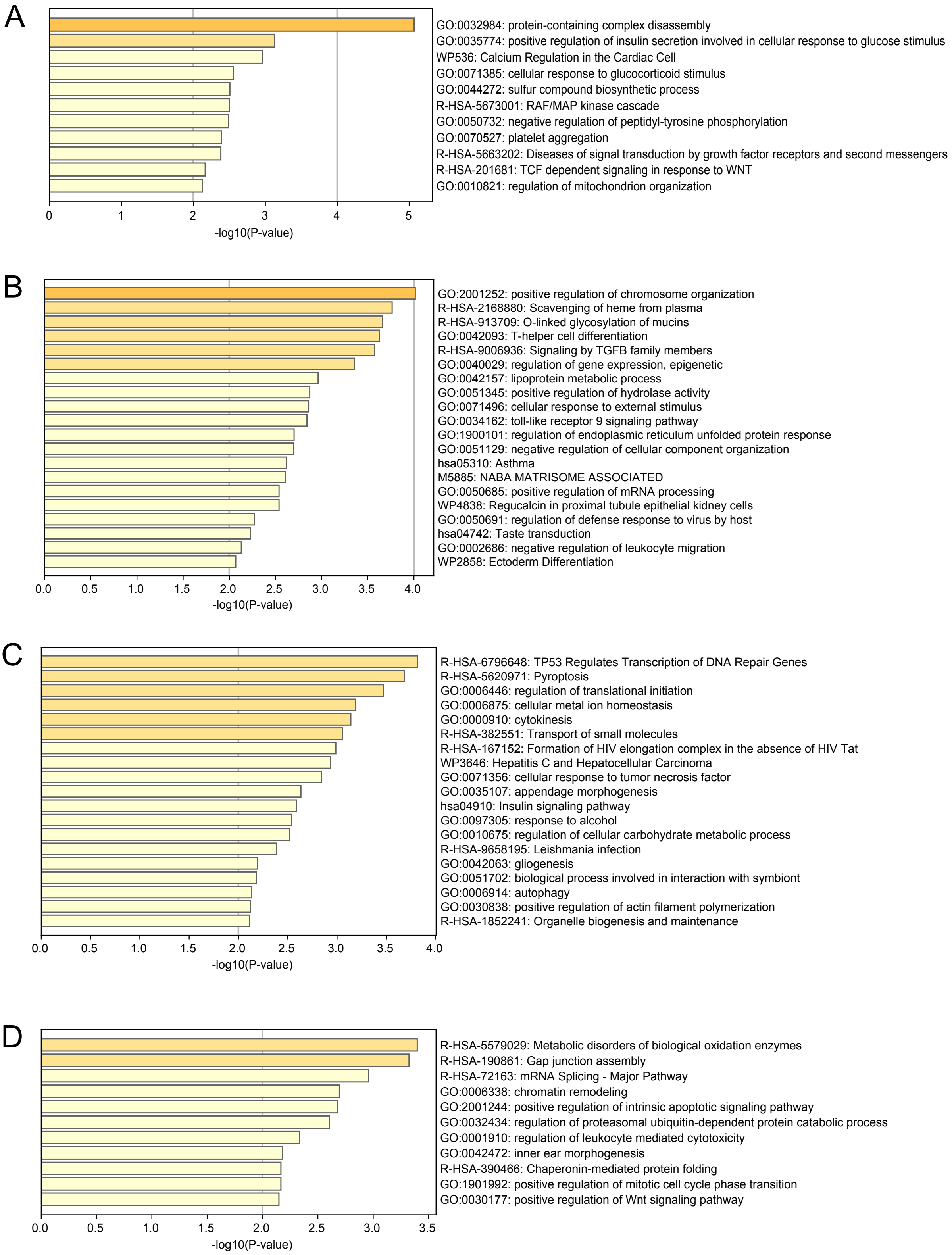

Fig. S24. Functional categories enriched from the differential gene set in each of M.Yunnan.West.

Functional categories enriched from the differential gene sets of (A) ACH, (B) DAI, (C) DEA, and (D) JIP, using *metascape* (https://metascape.org). Functional category with $-\log_{10}(P\text{-value}) \geq 2$ was displayed as enriched term across input gene set. Similar functional categories were classified into one group and the category with highest logarithm value was shown. The other functional categories of each group are listed in Table S8.

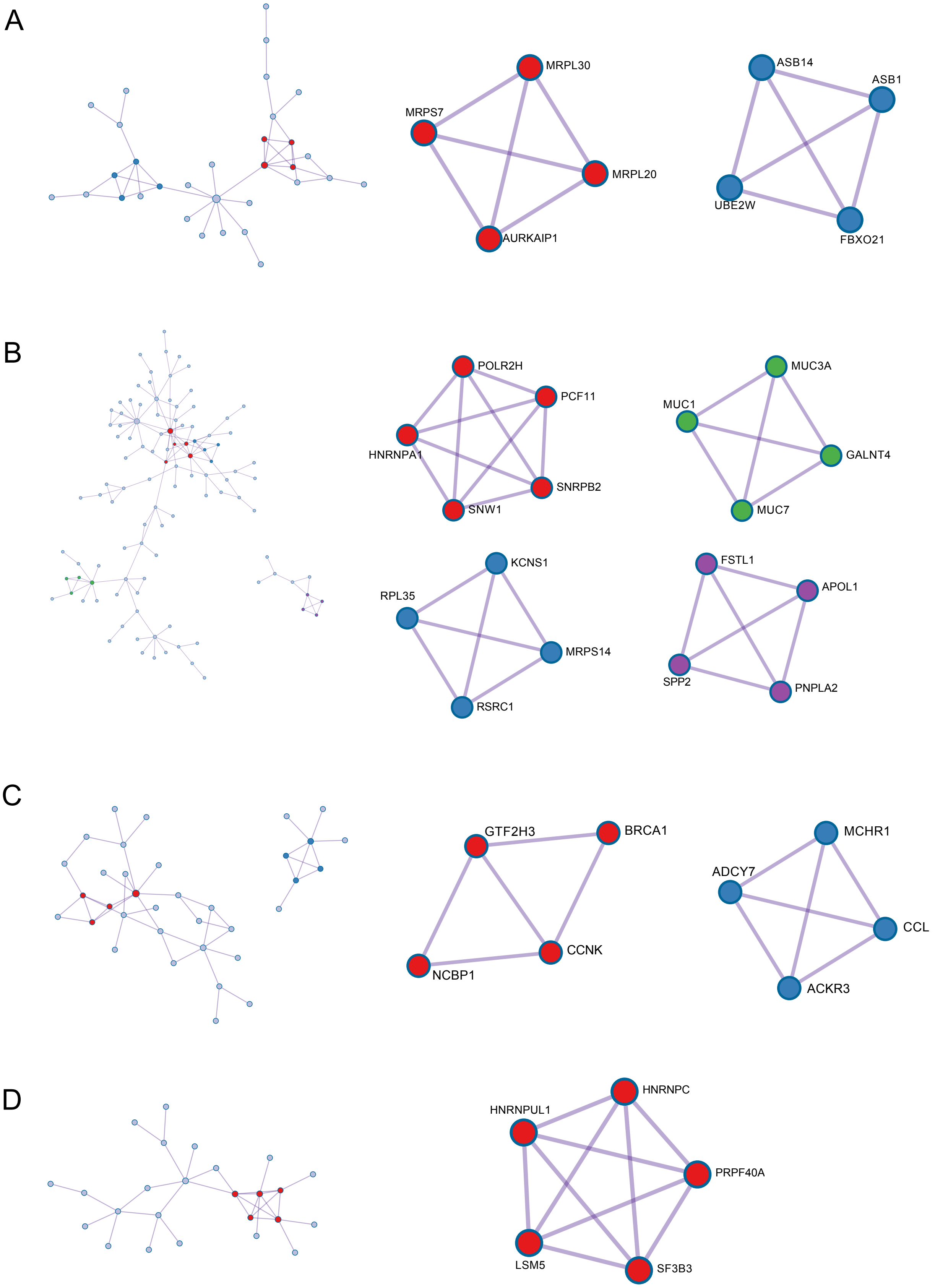

Fig. S25. Protein-protein interaction (PPI) identified from the differential gene set in each of M.Yunnan.West.

Protein network analysis identified from the differential gene sets of (A) ACH, (B) DAI, (C) DEA, and (D) JIP, using *metascape* (https://metascape.org). For each subplot, the whole PPI network harboring differential genes and the local PPI network are displayed in the left and right, respectively.

Table S2. Information of populations in Global Panel C.

Description of the populations in Global Panel C, including sample sizes, geographic locations, and data sources.

Region	Population	Macro-population	Sample Size	Latitude	Longitude	Altitude	District	Source
East Asia (China)	Achang	M.Yunnan.West	65	24.51	98.12	1439	Yunnan	This Study
	Dai	M.Yunnan.West	52	24.21	98.2	420	Yunnan	
	Deang	M.Yunnan.West	65	24.17	98.16	1286	Yunnan	
	Jingpo	M.Yunnan.West	60	24.15	98.17	1711	Yunnan	
	Han	HAN.North	50	36	117	146	Shandong	HuaBiao Project
	Han	HAN.North	50	34.5	113	56	Henan	
	Han	HAN.South	50	31	121	71	Shanghai	
	Han	HAN.South	50	33	119	2	Jiangsu	
	Han	HAN.South	50	29	120	42	Zhejiang	
	Han	HAN.South	50	31	116	89	Anhui	
	Tibetan	M.Highland	33	29.62	91.56	3605	Tibet	Lu et al 2016
	Naxi	M.Highland	8	26.5	99.5	2530	Yunnan	HGDP
	Tu	M.Highland	10	36	101	2490	Qinghai	
	Yi	M.Highland	10	28	103	2456	Sichuan	
	Lahu	M.South	8	23	100	1565	Yunnan	
	Miao	M.South	10	28	108	546	Guizhou	
	She	M.South	10	27	119	103	Fujian	
	Tujia	M.South	9	29	109	198	Hubei	
	Dai	M.Yunnna.West	9	22	101	572	Yunnan	
	Han	HAN.North	10	32.5	109	80	-	
		HAN.South	33				-	
Mainland Southeast Asia	Cambodian	MSEA	9	12	105	108	-	
	Malay	MSEA	25	4.61	101.69	131	-	Mörseburg et al 2016
	Burmese	MSEA	20	24.02	96.36	324	-	
	Vietnamese	MSEA	18	21.16	105.61	18	-	

Table S5. Demographic parameters of 5-population model with 95% CI estimated by *dadi*.

Parameters were estimated using the mutation rate of 2.5×10^{-8} per generation per site and a generation time of 25 years. T1: Timing of the split between (ACH, JIP) and ((DAI, DEA), HAN); T2: Timing of the split of ACH and JIP; T3: Timing of the split between (DAI, DEA) and HAN; Timing of the split between DAI and DEA; NA: Ancestral effective population size; NB: Effective population size of the ancestor of ACH and JIP; NC: Effective population size of the ancestor of DAI, DEA, and HAN; ND: Effective population size of the ancestor of DAI and DEA. N1: Effective population size of ACH; N2: Effective population size of JIP; N3: Effective population size of DAI; N4: Effective population size of DEA; N5: Effective population size of HAN.

Parameter	Estimation	95% CI	
		Lower	Upper
Log-likelihood	-4366.08	-	-
Theta	3057.56	2904.20	3210.64
T1	12839.08	12096.89	13480.89
T2	3771.79	3582.89	3960.49
T3	8905.15	8458.89	9349.17
T4	6988.21	6638.60	7237.43
NA	6556.37	6227.99	6884.54
NB	8796.42	8356.62	9235.66
NC	9941.27	9544.60	12438.59
ND	12011.61	11411.30	12611.67
N1	6721.60	3535.08	3907.11
N2	6248.29	3751.51	4145.91
N3	19097.40	18146.35	20052.97
N4	3479.34	3305.24	3652.86
N5	25644.43	24364.64	26925.68
Mis	0.0793	0.0780	0.0813

Table S6. Number of novel variants of annotation categories defined by *VEP*.

Annotation categories for novel variants of each M.Yunnan.West and HAN were defined by VEP. The number of annotation categories of these novel variants excluding singletons are shown in the table.

Category		Population					Total
		ACH	DAI	DEA	JIP	HAN	
Loss-of-function	missense variant (SIFT<0.05 and PolyPhen>0.446)	326	122	349	282	77	1156
	start lost	2	2	2	6	0	12
	stop gained	32	7	20	19	10	88
	stop lost	1	1	1	1	2	6
	splice acceptor variant	1	1	5	5	0	12
	splice donor variant	3	1	0	2	1	7
missense variant (SIFT≥0.05 and PolyPhen≤0.446)		567	193	699	533	153	2145
synonymous variant		453	125	512	395	84	1569
Non-coding	non-coding transcript exon variant	99	40	110	85	44	378
	mature miRNA variant	0	2	0	0	0	2
	intron variant	1151	383	1314	1012	421	4281
UTR	3 prime UTR variant	207	63	260	179	82	791
	5 prime UTR variant	59	28	80	52	20	239
intergenic variant		88	33	114	83	21	339
upstream gene variant		85	35	114	89	31	354
downstream gene variant		151	63	161	126	59	560
regulatory region variant		15	4	13	6	4	42
Total		3240	1103	3754	2875	1009	11981
