# Supplementary material for: Genetic evidence of tri-genealogy hypothesis on the origin of ethnic minorities in Yunnan
Source: BMC Biol. 2022 Jul 21;20:166. doi: 10.1186/s12915-022-01367-3 (PMC9306206; doi:10.1186/s12915-022-01367-3)
Supplement: Supplementary file 2 — Additional file 2. Introduction of studied ethnic minorities. [file 12915_2022_1367_MOESM2_ESM.docx]

**Additional file 2 - Introduction of the studied ethnic minorities**

**Achang and Jingpo**

The Achang (ACH) and Jingpo (JIP) populations are ethnic groups that mainly live in the Western Yunnan boarding with Northeastern Myanmar. The language of ACH and JIP belongs to the Tibeto-Burman language family while they also speak Mandarin or Dai languages due to neighboring with the Han Chinese (HAN) and Dai (DAI) populations. The earliest historical record in China of ACH and JIP could be traced to the Tang Dynasty (618–907 AD) and they were collectively called the Xunchuan people before 16^th^ century [1]. From the Ming Dynasty (1368–1644 AD), the ACH and JIP were largely under the control of DAI people in accordance with the Tusi system in ancient China [2, 3]. The previous ethnological and linguistic studies proposed that ACH and JIP were derived from the Upper-Middle Yellow River that was dominated by Di-Qiang populations [4]. They gradually migrated through Jinsha River and Nu River, and finally settled down in current Western Yunnan [2, 5]. Thus, they were considered as the descendant of Di-Qiang lineage with close genetic relationship.

However, the cultural conventions of ACH and JIP could be different. ACH prefer to preserve and transmit their history and folk tales by oral stories, ballad, and antiphonal singing [1]. In addition, ACH believe multiple religions due to the influence of diverse culture such as Theravada Buddhism of Dai people. As for JIP, most people remain animist in faith like Shamanism [5]. Interestingly, JIP people in one tribe would marry with another tribe since JIP always try to avoid intermarriage. However, since there are many dialect branches in Jingpo language, Jingpo people to be married from different tribes may speak different dialect and they will retain their own dialects even after marriage [1].

**Dai**

The DAI are a relatively large and prosperous ethnic group distributed in Southern and Western Yunnan. They DAI language belong to the Tai-Kadai language family that is mainly used by the Bai-Yue populations. The earliest DAI was distributed in the upper Yangtze River. During the Han (206 BC – 220 AD) Dynasty, the DAI started to be affiliated with the HAN with trade and culture communications [3]. From the Yuan Dynasty (1271–1368 AD), the DAI were appointed to become the head in Southwestern China to rule over other ethnic groups. The DAI were benefited from this neo-feudal system called Tusi to have a higher social status than surrounding ethnic groups along the ancient Chinese history [2].

The DAI had a well-developed system of agriculture and were good at the cultivation of rice. They also prefer living in bamboo houses with green trees and eating foods with bitterness due to living in the hot and humid environment [1]. The DAI had a prosperous and distinctive culture. For example, the DAI had their own calendar and celebrate the day of New Year as Water-Splashing Festival (also known as Songkran) [1, 2]. The cultural music and art of DAI such as Elephant Foot Drums and Peacock Dance were famous in the world. Most of DAI people are the followers of Theravada Buddhism. The religion of DAI has influenced surrounding ethnic groups that were once under the control of DAI.

**Deang**

The Deang (DEA) people live neighboring with DAI, JIP, and HAN in Western Yunnan, they are one of the three ethnic groups speaking Austroasiatic languages in China. The earliest historical records of DEA could be traced back to the Qin Dynasty (221–207 BC), when they were regarded as the native population called Pu people in Western Yunnan [2, 6]. The DEA and other Austroasiatic-speaking populations were collectively called Bai-Pu (Bai means the hundreds of) before the Qing Dynasty. Similar to the ACH and JIP, the DEA were also under the control of DAI before 19^th^ century [2, 3]. As the aboriginal population in Western Yunnan though, the DEA gradually moved to mountainous area with barren land in Western Yunnan due to the control of the DAI and the migration of the ACH and JIP.

The cultural customs of the DEA are mostly close to those of the DAI since they were under the long-term control of the DAI. For example, the DEA follow the DAI to practice the Theravada Buddhism and celebrate the Water-Splashing Festival [1, 5]. The DEA also retained their own culture customs like tea culture. The DEA grow and drink tea for a long history since they believe tea carries their good wishes and welcome [1]. The Sour Tea is one of the reprehensive teas in DEA tea culture.

**References**

1. Liu J. Nationalities in Southwest China: <http://www.kepu.net.cn/english/nationalitysw>. Accessed Apr 2022.

2. Cang M. Study on the migration culture of the ethnic groups in Yunan: Yunnan Nationality Press; 1997.

3. Wang Z. History of nationalities in China.: China Social Science Press; 1994.

4. Xu B, Guo J, Huang Y, Chen X, Deng X, Wang CC. The paternal genetic structure of Jingpo and Dai in southwest China. Ann Hum Biol. 2019;46(3):279-83.

5. Miller L. South of the Clouds: Tales from Yunnan: University of Washington Press; 2017.

6. Olson J. An Ethnohistorical Dictionary of China: Greenwood Publishing Group; 1998.
